# Supplementary material for: Nonlinear Effects Induced by Interactions among Functional Groups of Bacteria and Fungi Regulate the Priming Effect in Malagasy Soils
Source: Microorganisms. 2023 Apr 23;11(5):1106. doi: 10.3390/microorganisms11051106 (PMC10222199; doi:10.3390/microorganisms11051106)
Supplement: Supplementary file 1 [file microorganisms-11-01106-s001.zip › microorganisms-2327025-supplementary.pdf]

Table S1: Physico-chemical properties of soil samples

SAMPLE	long	lat	alt	MAP	MAT	BD	clay	silt	sand	pH	CEC	Al_ox	Fe_ox	total_C	total_N	total_P	soil_CN	CP_soil	available_P	mic_P
	DD	DD	m	mm.an-1	°C.an-1	g.cm-3	%	%	%		cmol+ .kg-1	mg.g-1 sol	mg.g-1 sol	mg.g-1 sol	mg.g-1 sol	mg.g-1 sol			mg.kg-1 sol	mg.kg-1 sol
**RN34_1**	46,187997	-19,587120	915	1504	21,9	1,52	29,04	4,42	66,54	4,72	3,92	1,57	0,91	15,10	1,00	0,2734	15,03	55,22	1,21	1,35
**RN34_2**	46,082402	-19,598820	1031	1492	21,9	1,30	39,46	4,91	55,63	5,83	4,22	2,39	2,35	28,05	1,94	0,4491	14,44	62,44	0,46	2,83
**RN34_3**	45,978408	-19,639120	813	1465	22,7	1,22	47,18	7,05	45,77	6,18	3,04	1,93	1,71	11,47	0,93	0,2313	12,32	49,60	0,14	1,06
**RN34_4**	45,978408	-19,639120	813	1465	22,7	1,22	47,18	7,05	45,77	6,18	3,04	1,93	1,71	11,47	0,93	0,2313	12,32	49,60	0,17	1,41
**RN34_6**	45,787117	-19,648419	680	1430	23,4	1,23	52,93	7,48	39,59	5,53	1,48	1,40	1,96	18,07	1,16	0,2539	15,57	71,20	0,49	4,26
**RN34_8**	46,337189	-19,577919	945	1508	21,8	1,42	42,21	8,48	49,31	5,45	1,17	1,56	0,62	15,55	1,17	0,3251	13,28	47,81	0,42	3,10
**RN34_9**	46,483831	-19,622050	1038	1512	20,9	1,42	61,62	0,98	37,39	5,39	3,40	1,94	1,98	16,14	1,26	0,5197	12,76	31,06	0,71	2,88
**RN34_10**	46,617275	-19,721217	1128	1504	20,1	1,19	41,70	12,13	46,16	5,55	2,85	2,18	1,31	18,80	1,26	0,3917	14,92	48,01	0,51	2,46
**RN34_11**	46,617275	-19,721217	1128	1504	20,1	1,19	41,70	12,13	46,16	5,55	2,85	2,18	1,31	18,80	1,26	0,3917	14,92	48,01	0,33	2,42
**RN34_12**	46,617275	-19,721217	1128	1504	20,1	1,19	41,70	12,13	46,16	5,55	2,85	2,18	1,31	18,80	1,26	0,3917	14,92	48,01	0,26	2,20
**RN34_13**	46,763767	-19,807017	1298	1479	18,6	1,13	55,48	5,64	38,88	5,29	2,15	3,96	1,78	24,68	1,70	0,6190	14,48	39,87	0,53	2,77
**RN4_36**	46,911856	-16,970067	64	1668	26,8	1,52	16,00	11,84	72,15	6,31	8,29	1,02	1,29	3,74	0,32	0,2991	11,85	12,50	0,33	0,47
**RN4_37**	46,806661	-17,116664	214	1694	26,7	1,32	38,36	15,62	46,02	6,44	3,45	0,61	2,24	7,66	0,48	0,2408	16,06	31,79	0,23	0,51
**RN4_38**	46,806661	-17,116664	214	1694	26,7	1,32	38,36	15,62	46,02	6,44	3,45	0,61	2,24	7,66	0,48	0,2408	16,06	31,79	0,39	1,39
**RN4_39**	46,806661	-17,116664	214	1694	26,7	1,32	38,36	15,62	46,02	6,44	3,45	0,61	2,24	7,66	0,48	0,2408	16,06	31,79	0,62	2,48
**RN4_40**	46,930556	-17,359758	394	1759	25,7	1,34	56,92	2,67	40,41	5,86	3,44	0,91	1,59	13,52	0,89	0,3577	15,24	37,79	0,10	1,17
**RN4_41**	46,987253	-17,486558	639	1776	24,0	1,35	47,37	3,13	49,50	5,61	2,52	1,64	2,12	17,41	1,29	0,3190	13,52	54,57	0,50	2,90
**RN4_42**	46,954956	-17,622056	655	1817	24,0	1,15	46,05	3,64	50,31	5,75	3,07	2,46	1,29	23,87	1,72	0,4109	13,87	58,08	0,44	2,97
**RN4_43**	46,954956	-17,622056	655	1817	24,0	1,15	46,05	3,64	50,31	5,75	3,07	2,46	1,29	23,87	1,72	0,4109	13,87	58,08	0,17	1,13
**RN4_44**	46,954956	-17,622056	655	1817	24,0	1,15	46,05	3,64	50,31	5,75	3,07	2,46	1,29	23,87	1,72	0,4109	13,87	58,08	0,74	3,76
**RN4_46**	47,104347	-17,908650	1477	1500	18,5	1,18	39,99	6,35	53,66	5,50	1,05	5,12	6,94	23,92	1,07	0,7240	22,42	33,03	0,08	0,91
**RN4_47**	47,152844	-18,033647	1558	1468	18,1	1,13	47,55	3,32	49,13	5,37	1,75	6,71	1,51	36,58	2,08	0,3418	17,58	107,03	0,63	3,61
**RN4_48**	47,152844	-18,033647	1558	1468	18,1	1,13	47,55	3,32	49,13	5,37	1,75	6,71	1,51	36,58	2,08	0,3418	17,58	107,03	1,07	6,34
**RN4_49**	47,152844	-18,033647	1558	1468	18,1	1,13	47,55	3,32	49,13	5,37	1,75	6,71	1,51	36,58	2,08	0,3418	17,58	107,03	0,92	5,31
**RN4_50**	47,215542	-18,131644	1556	1435	17,7	1,19	54,33	2,62	43,05	5,58	2,13	2,04	2,01	39,39	2,27	0,4752	17,36	82,89	0,59	5,66
**RN4_51**	47,142747	-18,265542	1295	1466	19,1	1,36	38,41	3,07	58,52	5,36	3,09	3,23	0,53	22,59	1,08	0,3604	20,88	62,67	0,93	5,18
**RN4_52**	47,142747	-18,265542	1295	1466	19,1	1,36	38,41	3,07	58,52	5,36	3,09	3,23	0,53	22,59	1,08	0,3604	20,88	62,67	0,54	3,71
**RN4_53**	47,142747	-18,265542	1295	1466	19,1	1,36	38,41	3,07	58,52	5,36	3,09	3,23	0,53	22,59	1,08	0,3604	20,88	62,67	0,56	4,23
**RN4_54**	47,137547	-18,395742	1308	1450	19,1	1,31	43,09	4,04	52,87	5,22	2,19	1,43	1,05	25,63	1,43	0,3743	17,94	68,48	0,99	6,91
**RN4_55**	47,199544	-18,541639	1421	1402	18,3	1,22	42,22	5,23	52,55	5,17	3,59	1,43	3,19	36,82	1,86	0,6470	19,79	56,91	0,84	6,62
**RN4_56**	47,293339	-18,668636	1308	1363	18,9	1,32	40,67	8,32	51,02	5,16	0,80	1,73	1,40	18,13	1,09	0,4561	16,67	39,75	0,53	1,92
**RN4_57**	47,393133	-18,778133	1295	1327	18,8	1,21	41,78	2,42	55,80	5,62	1,24	1,68	1,26	28,08	1,60	0,4794	17,56	58,56	1,33	8,76
**RN7_75**	45,477339	-22,537167	802	783	22,0	1,53	18,64	23,69	57,66	5,62	3,20	0,88	0,91	14,70	0,85	0,2900	17,24	50,70	2,80	3,63
**RN7_76**	45,670431	-22,498169	955	790	21,2	1,46	21,84	5,93	72,23	5,27	0,99	1,18	0,42	15,85	0,85	0,2604	18,58	60,87	1,20	2,93
**RN7_77**	45,854019	-22,429669	1031	798	20,7	1,50	23,82	3,91	72,27	4,75	1,99	0,62	0,72	17,10	0,86	0,2731	19,91	62,63	0,87	1,48
**RN7_78**	45,854019	-22,429669	1031	798	20,7	1,52	33,55	2,7	63,75	4,75	1,95	0,62	0,72	12,35	0,69	0,1957	17,84	63,12	0,85	1,32
**RN7_79**	45,854019	-22,429669	1031	798	20,7	1,49	21,39	15,33	63,28	4,75	2,85	0,62	0,72	10,73	0,68	0,2081	15,88	51,57	1,28	2,24
**RN7_80**	46,027111	-22,400969	1123	819	20,2	1,20	27,78	4,38	67,84	4,86	3,04	0,58	0,50	19,95	1,16	0,5897	17,22	33,83	1,45	7,72
**RN7_81**	46,291797	-22,298769	747	779	21,7	1,37	33,71	3,65	62,64	4,98	4,99	0,89	0,36	25,39	1,22	0,3281	20,75	77,39	3,53	3,02
**RN7_82**	46,585981	-21,961678	774	926	21,1	1,49	32,34	6,70	60,96	5,75	3,98	1,57	0,63	18,00	1,08	0,5847	16,75	30,79	2,72	2,70
**RN7_83**	46,738272	-21,931878	1014	990	19,6	1,12	42,80	12,91	44,29	5,94	2,20	1,96	1,34	16,22	1,37	0,4068	11,84	39,88	0,77	2,70
**RN7_84**	46,738272	-21,931878	1014	990	19,6	1,31	52,00	11,55	36,45	5,94	3,09	1,96	1,34	15,57	1,09	0,4664	14,33	33,38	0,82	2,74
**RN7_85**	46,738272	-21,931878	1014	990	19,6	1,23	46,10	6,89	47,00	5,94	2,98	1,96	1,34	17,45	1,13	0,4385	15,41	39,80	1,20	3,63
**RN7_87**	46,952961	-21,774081	1098	1106	18,9	1,44	42,67	12,37	44,96	5,86	3,74	1,05	1,21	14,48	1,08	0,3092	13,47	46,83	0,54	2,05
**RN7_88**	47,019431	-21,583611	1174	1171	18,6	1,26	56,40	3,48	40,12	4,93	2,52	1,56	1,43	24,58	1,36	0,3450	18,03	71,24	0,72	4,53
**RN7_89**	47,123839	-21,445450	1135	1271	18,6	1,26	58,31	3,71	37,98	4,77	2,33	1,52	2,03	22,92	1,41	1,3371	16,29	17,14	0,31	5,25
**RN7_90**	47,174550	-21,358586	1140	1327	18,6	1,16	39,67	5,49	54,84	5,53	1,49	3,88	1,73	28,58	1,64	0,4845	17,40	59,00	0,45	2,43
**RN7_91**	47,174550	-21,358586	1140	1327	18,6	1,12	52,99	4,03	42,98	5,53	2,66	3,88	1,73	30,02	1,69	0,4040	17,71	74,31	0,62	3,93
**RN7_92**	47,174550	-21,358586	1140	1327	18,6	1,13	47,33	4,42	48,25	5,53	3,14	3,88	1,73	21,59	1,21	0,3121	17,89	69,16	0,40	2,25
**RN7_93**	47,244544	-21,150792	1238	1368	17,9	1,10	60,95	3,68	35,37	4,95	2,10	2,15	2,28	41,61	2,26	1,5117	18,38	27,53	1,13	10,19
**RN7_94**	47,182350	-21,037592	1246	1333	17,8	1,29	45,67	4,03	50,30	5,89	2,13	1,97	0,78	22,82	1,31	0,2639	17,48	86,49	0,68	4,91
**RN7_95**	47,137050	-20,893894	1443	1345	16,9	1,12	65,17	5,19	29,64	5,98	6,27	1,28	2,02	43,82	2,65	0,4822	16,55	90,88	1,43	20,98
**RN7_96**	47,209847	-20,600300	1534	1418	16,4	1,30	46,01	3,68	50,31	5,73	2,41	2,12	0,77	35,42	1,84	0,2832	19,28	125,11	1,52	12,70
**RN7_97**	47,231631	-20,461200	1343	1491	17,4	0,72	29,16	8,17	62,67	5,69	1,96	1,76	3,90	41,06	2,20	1,0145	18,68	40,47	0,60	7,96
**RN7_98**	47,231631	-20,461200	1343	1491	17,4	1,15	67,04	5,61	27,35	5,69	2,93	1,76	3,90	39,93	2,25	0,9894	17,76	40,35	0,53	7,01
**RN7_99**	47,231631	-20,461200	1343	1491	17,4	1,14	71,62	5,51	22,86	5,69	3,05	1,76	3,90	27,64	1,91	0,5560	14,47	49,71	0,47	11,29
**RN7_100**	47,125950	-20,322406	1399	1441	17,4	1,28	58,73	4,5	36,77	6,29	3,17	2,33	1,08	27,84	1,62	0,3808	17,24	73,11	0,94	12,16

Abbreviations: long: longitude; lat: latitude; alt: altitude ; MAP: Mean Annual Precipitation; MAT: Mean Annual Temperature; BD: Bulk density; Al ox: Aluminium oxidable; Fe ox: Iron oxidable, total C: total carbon; total N: total nitrogen; total P: total phosphorus; Soil_CN: total C on total N ratio; Soil_CN: totl C on total P ratio; available P: extractable phosphorus by the resin method; mic P: microbial biomass phosphorus.

Table S2

Bacteria		Fungi	
Family	Phylum	Family	Phylum
Acetobacteraceae	α-Proteobacteria	Agaricaceae	Basidiomycota
Acidimicrobineae incertae sedis	Actinobacteria	Agyriaceae	Ascomycota
Acidobacteria-Gp1	Acidobacteria	Ajellomycetaceae	Ascomycota
Acidobacteria-Gp13	Acidobacteria	Ambisporaceae	Glomeromycota
Acidobacteria-Gp2	Acidobacteria	Auriculariaceae	Basidiomycota
Acidobacteria-Gp3	Acidobacteria	Bionectriaceae	Ascomycota
Acidobacteria-Gp4	Acidobacteria	Boletaceae	Basidiomycota
Acidobacteria-Gp5	Acidobacteria	Bulgariaceae	Ascomycota
Acidobacteria-Gp6	Acidobacteria	Chaetomiaceae	Ascomycota
Acidobacteria-Gp7	Acidobacteria	Clavicipitaceae	Ascomycota
Actinospicaceae	Actinobacteria	Coniochaetaceae	Ascomycota
Armatimonadaceae	Armatimonadetes	Coniophoraceae	Basidiomycota
Bacillaceae	Firmicutes	Cordycipitaceae	Ascomycota
Bdellovibrionaceae	δ-Proteobacteria	Corticiaceae	Basidiomycota
Beijerinckiaceae	α-Proteobacteria	Davidiellaceae	Ascomycota
Bradyrhizobiaceae	α-Proteobacteria	Didymellaceae	Ascomycota
Burkholderiaceae	β-Proteobacteria	Endogonaceae	Mucuromycota
Catenulisporaceae	Actinobacteria	Entolomataceae	Basidiomycota
Caulobacteraceae	α-Proteobacteria	Environmental	-
Chitinophagaceae	Bacteroïdetes	Glomeraceae	Glomeromycota
Chloroplast	Eucaryotes	Gomphaceae	Basidiomycota
Chthonomonadaceae	Armatimonadetes	Helotiaceae	Ascomycota
Comamonadaceae	β-Proteobacteria	Herpotrichiellaceae	Ascomycota
Conexibacteraceae	Actinobacteria	Hyaloscyphaceae	Ascomycota
Coxiellaceae	γ-Proteobacteria	Hypocreaceae	Ascomycota
Cyanobacteria.Chloroplast-F1	Cyanobacteria	Kickxellaceae	Kickxellomycotina
Cystobacteraceae	δ-Proteobacteria	Lycoperdaceae	Basidiomycota
Fervidicoccaceae	Crenarchaetoa	Lyophyllaceae	Basidiomycota
Flavobacteriaceae	Bacteroïdetes	Magnaporthaceae	Ascomycota
Gemmatimonadaceae	Gemmatimonadetes	Marasmiaceae	Basidiomycota
Geodermatophilaceae	Actinobacteria	Massariaceae	Ascomycota
Haliangiaceae	δ-Proteobacteria	Monoblepharidaceae	Chytridiomycota
Hyphomicrobiaceae	α-Proteobacteria	Montagnulaceae	Ascomycota
Ktedonobacteraceae	Chloroflexi	Mortierellaceae	Mortierellomycota
Methylobacteriaceae	α-Proteobacteria	Mycosphaerellaceae	Ascomycota
Methylocystaceae	α-Proteobacteria	Myxotrichaceae	Ascomycota
Microbacteriaceae	Actinobacteria	Nectriaceae	Ascomycota
Micromonosporaceae	Actinobacteria	Ophiocordycipitaceae	Ascomycota
Mycobacteriaceae	Actinobacteria	Ophiostomataceae	Ascomycota
Nitrospiraceae	Nitrospira	Orbiliaceae	Ascomycota
Nocardioidaceae	Actinobacteria	Pezizaceae	Ascomycota
Oxalobacteraceae	β-Proteobacteria	Phaeosphaeriaceae	Ascomycota
Paenibacillaceae	Firmicutes	Plectosphaerellaceae	Ascomycota
Pasteuriaceae	Firmicutes	Pleosporaceae	Ascomycota
Phyllobacteriaceae	α-Proteobacteria	Pleurotaceae	Basidiomycota
Planctomycetaceae	Planctomycetes	Pluteaceae	Basidiomycota
Planococcaceae	Firmicutes	Pyronemataceae	Ascomycota
Polyangiaceae	δ-Proteobacteria	Rhizophydiaceae	Chytridiomycota
Pseudonocardiaceae	Actinobacteria	Sarcosomataceae	Ascomycota
Rhizobiales incertae sedis	Rhizobiales	Stereaceae	Basidiomycota
Rhodospirillaceae	α-Proteobacteria	Strophariaceae	Basidiomycota
Rubrobacteraceae	Actinobacteria	Teratosphaeriaceae	Ascomycota
Sinobacteraceae	γ-Proteobacteria	Trechisporaceae	Basidiomycota
Solirubrobacteraceae	Actinobacteria	Tremellaceae	Basidiomycota
Streptomycetaceae	Actinobacteria	Trichocomaceae	Ascomycota
Thermomonosporaceae	Actinobacteria	Tricholomataceae	Basidiomycota
Undefined	-	Tubeufiaceae	Ascomycota
Unknown	-	Unclassified	-
Xanthobacteraceae	α-Proteobacteria	Unknown	-
Xanthomonadaceae	γ-Proteobacteria	Ustilaginaceae	Basidiomycota

Table S3

Fobs.t7.summary

	substrate	duration of incubation	statistical model	p-value
bacteria	soil	7 days	*lm*	0.357
			fclust	0.675
		42 days	*lm*	0.933
			fclust	0.380
	straw	7 days	*lm*	0.736
			fclust	0.720
		42 days	*lm*	0.049
			fclust	0.234
	priming effect	7 days	*lm*	0.044
			fclust	0.360
		42 days	*lm*	0.118
			fclust	0.893
fungi	soil	7 days	*lm*	0.084
			fclust	0.483
		42 days	*lm*	0.118
			fclust	0.132
	straw	7 days	*lm*	0.373
			fclust	0.174
		42 days	*lm*	0.980
			fclust	0.331
	priming effect	7 days	*lm*	0.919
			fclust	0.981
		42 days	*lm*	0.657
			fclust	0.215
bacteria and fungi	soil	7 days	*fclust*	0.787
		42 days	*fclust*	0.186
	straw	7 days	*fclust*	0.216
		42 days	*fclust*	0.386
	priming effect	7 days	*fclust*	0.049
		42 days	*fclust*	0.832

Jaillard, B.; Razanamalala, K.; Violle, C.; Bernard, L. Nonlinear Effects Induced by Interactions among Functional Groups of Bacteria and Fungi Regulate the Priming Effect in Malagasy Soils. *Microorganisms.*

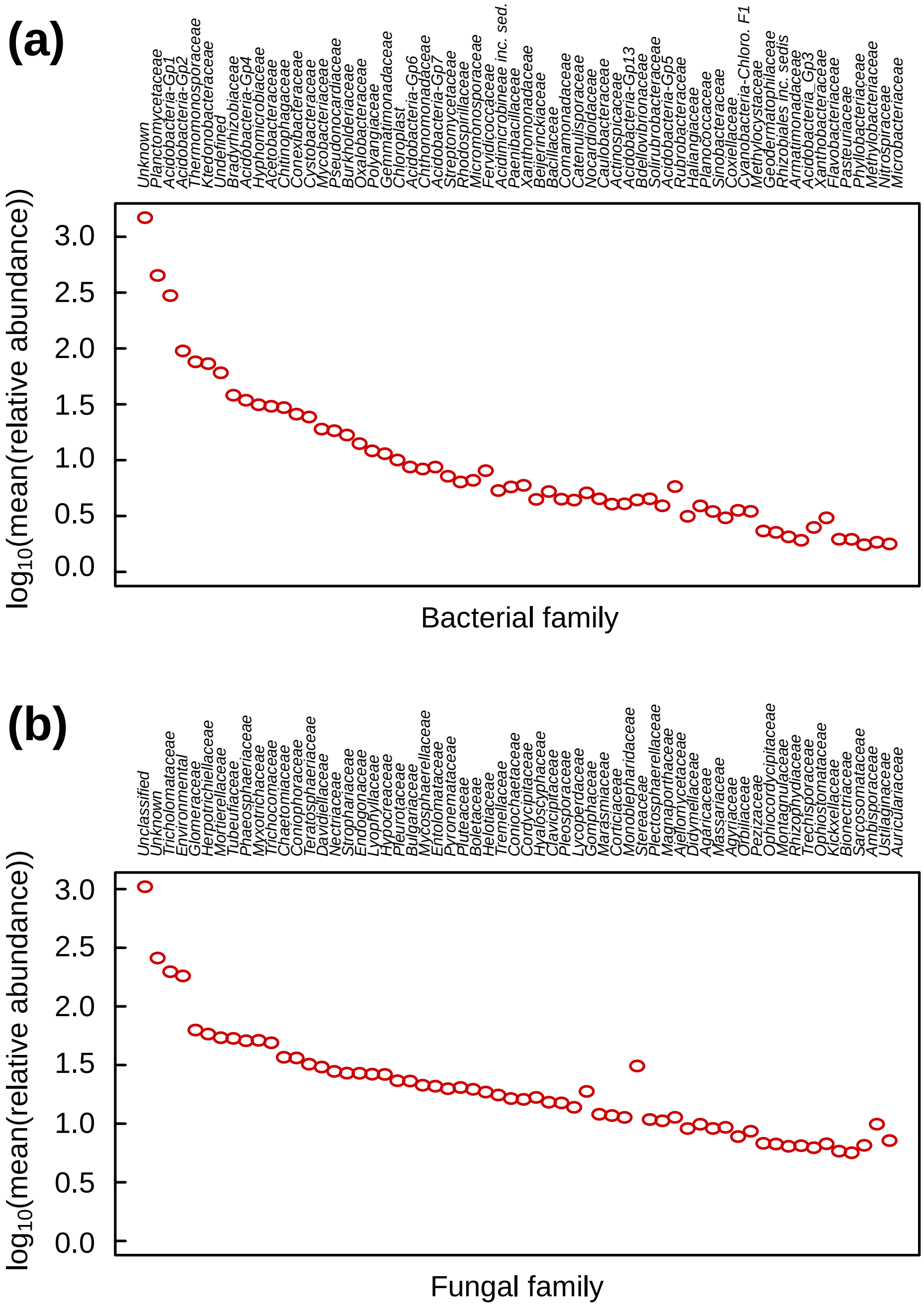

Figure S1. Relative abundance of bacterial and fungal families. (**a**) Bacteria. (**b**) Fungi. Families are sorted by decreasing sum of reads in the dataset.

Jaillard, B.; Razanamalala, K.; Violle, C.; Bernard, L. Nonlinear Effects Induced by Interactions among Functional Groups of Bacteria and Fungi Regulate the Priming Effect in Malagasy Soils. *Microorganisms.*

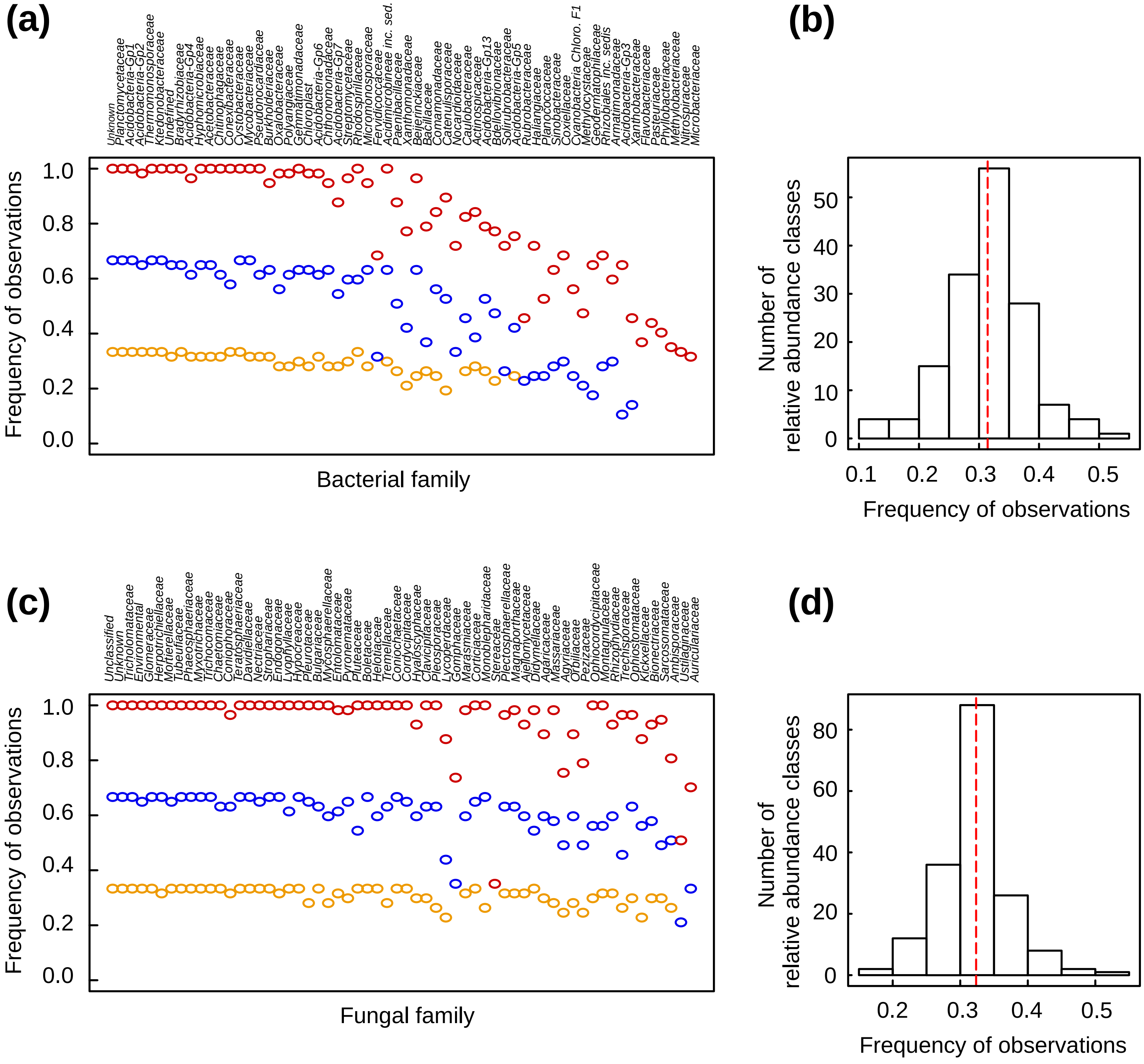

Figure S2. Conversion of abundances into classes of relative abundance for clustering analysis. (**a**) and (**b**) Bacteria. (**c**) and (**d**) Fungi. (**a**) and (**c**). Segmentation of the relative abundance distribution of each family into relative abundance classes. (**b**) and (**d**). Distribution of observation frequencies of the relative abundance classes obtained. The target frequency is 0.30. The median of the frequencies obtained is 0.322 and 0.333, for bacteria and fungus relative abundances, respectively. Families are sorted by decreasing sum of reads in the dataset, from left to right of the figure.

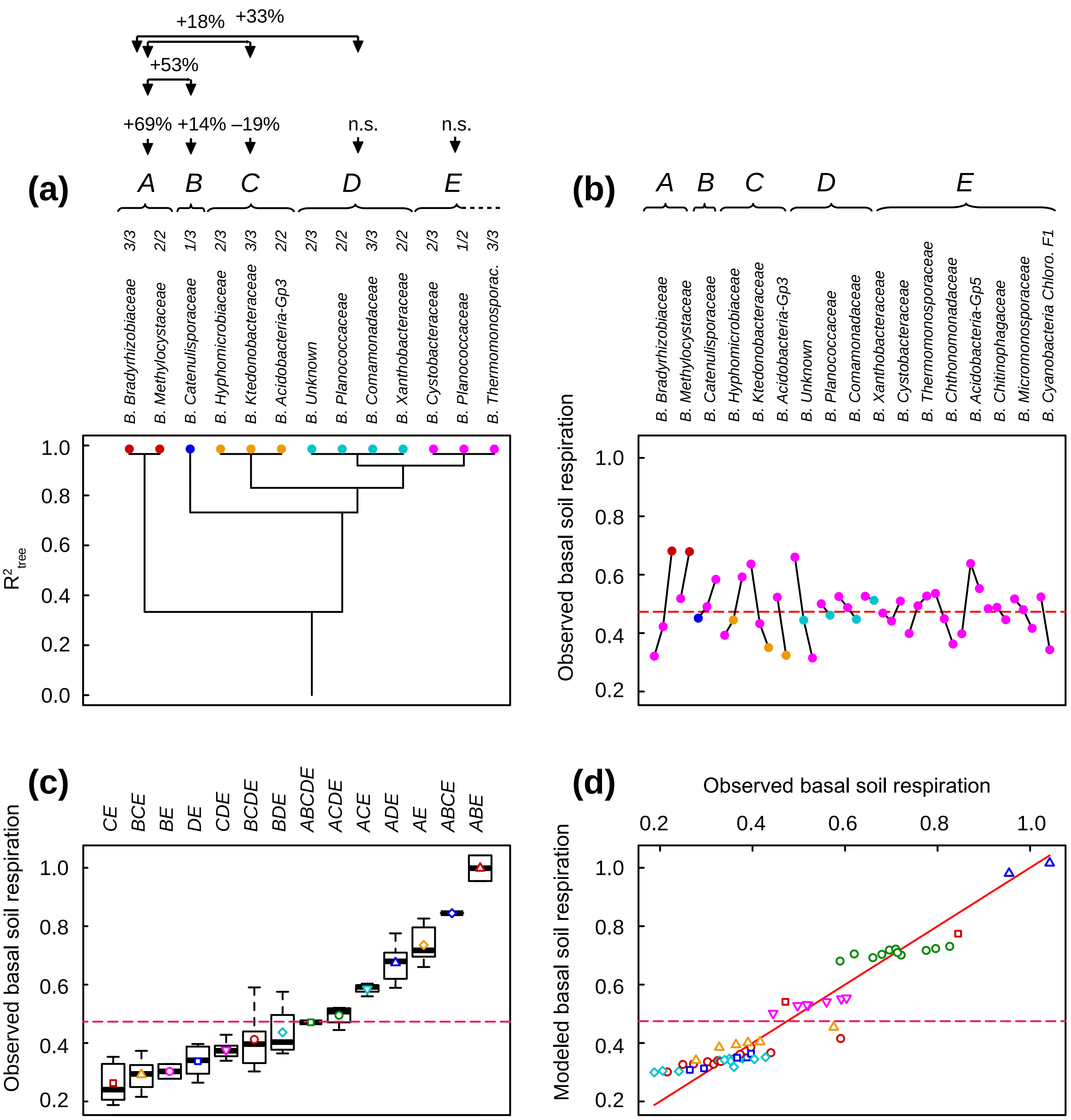

Figure S3. Clustering analysis of basal soil respiration after seven days of incubation, explained by the relative abundance classes of bacterial families in soil. (**a**) Clustering tree of classes of family relative abundance. The functional groups, and the classes of family relative abundance inside each functional group, are sorted from left to right by decreasing effect on the basal soil respiration. Each functional group has a color attributed: red for A, dark blue for B, yellow for C, light blue for D, and pink for E. The above box reports the significant effects (at $p < 0.001$) of the different functional groups and their interactions. (**b**) Mean observed basal soil respiration of soils containing a given class of family relative abundance. The linked points correspond to the different classes of increasing relative abundance (from left to right) for each family. The used symbols are the same as those used in (**a**). (**c**) Boxplots of the observed basal soil respiration sorted by assembly motifs, i.e., combinations of functional groups. The used symbols are the same as those used in (**b**). (**d**) Modeled versus observed soil property. Different symbols correspond to different assembly motifs, i.e., combinations of functional groups. The red line is the bisector. (**b**), (**c**) and (**d**) The dotted line is the mean observed basal soil respiration.

Jaillard, B.; Razanamalala, K.; Violle, C.; Bernard, L. Nonlinear Effects Induced by Interactions among Functional Groups of Bacteria and Fungi Regulate the Priming Effect in Malagasy Soils. *Microorganisms.*

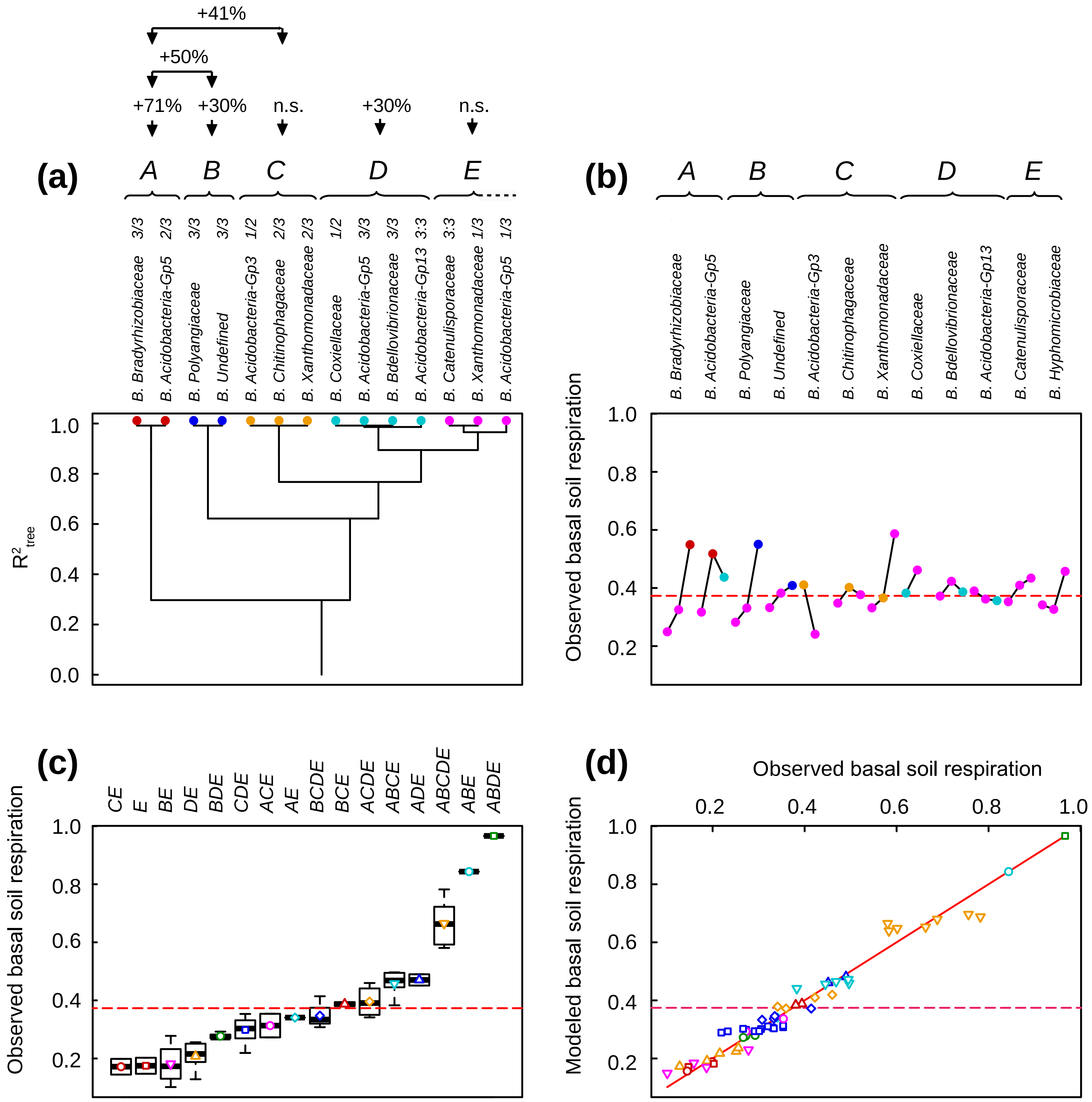

Figure S4. Clustering analysis of basal soil respiration after 42 days of incubation, explained by the relative abundance classes of bacterial families in soil. (**a**) Clustering tree of classes of family relative abundance. The functional groups, and the classes of family relative abundance inside each functional group, are sorted from left to right by decreasing effect on the basal soil respiration. Each functional group has a color attributed: red for A, dark blue for B, yellow for C, light blue for D, and pink for E. The above box reports the significant effects (at $p < 0.001$) of the different functional groups and their interactions. (**b**) Mean observed basal soil respiration of soils containing a given class of family relative abundance. The linked points correspond to the different classes of increasing relative abundance (from left to right) for each family. The used symbols are the same as those used in (**a**). (**c**) Boxplots of the observed basal soil respiration sorted by assembly motifs, i.e., combinations of functional groups. The used symbols are the same as those used in (**b**). (**d**) Modeled versus observed basal soil respiration. Different symbols correspond to different assembly motifs, i.e., combinations of functional groups. The red line is the bisector. (**b**), (**c**) and (**d**) The dotted line is the mean observed basal soil respiration.

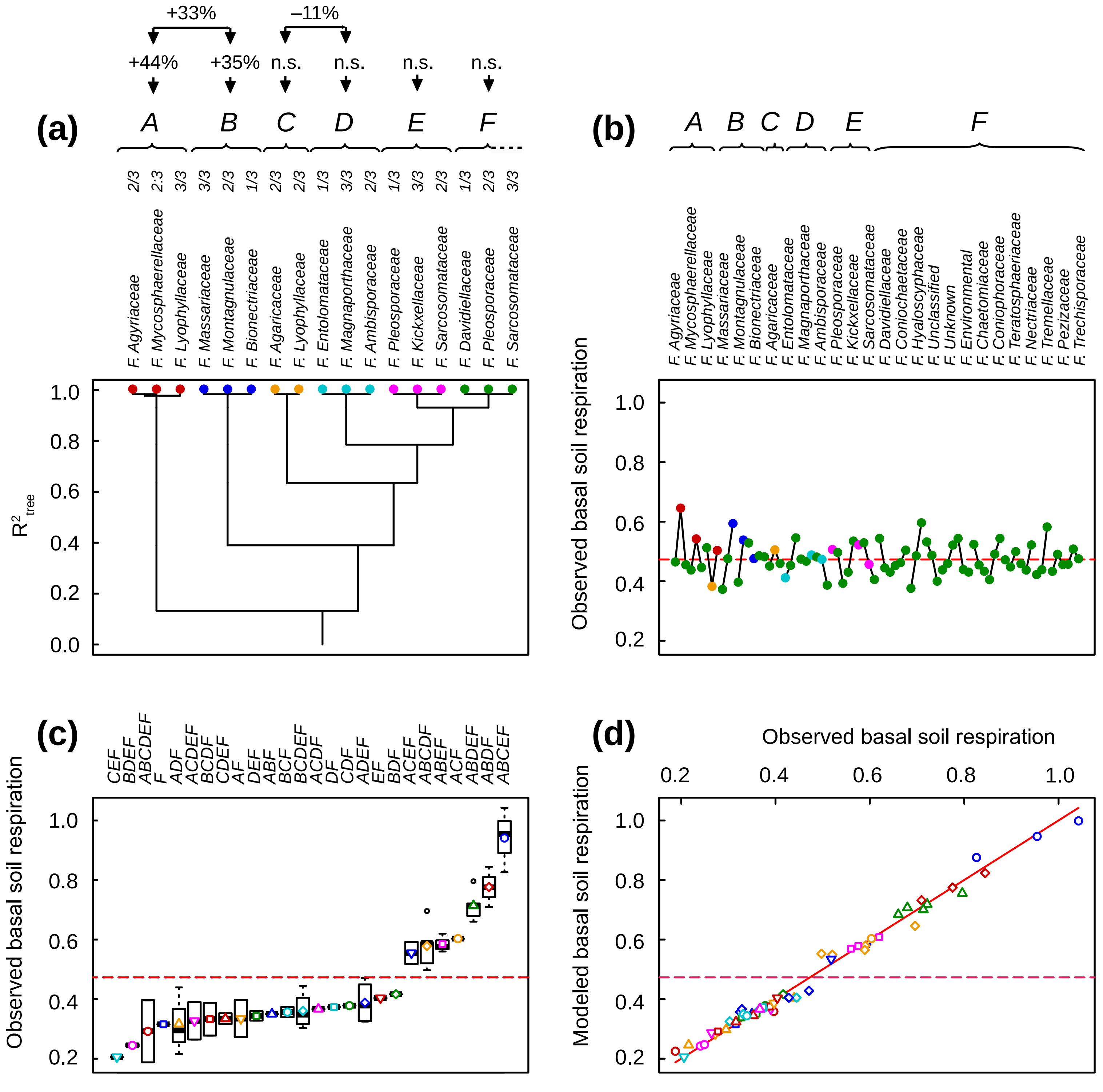

Figure S5. Clustering analysis of basal soil respiration after seven days of incubation, explained by the relative abundance classes of fungal families in soil. (**a**) Clustering tree of classes of family relative abundance. The functional groups, and the classes of family relative abundance inside each functional group, are sorted from left to right by decreasing effect on the basal soil respiration. Each functional group has a color attributed: red for A, dark blue for B, yellow for C, light blue for D, pink for E, and green for F. The above box reports the significant effects (at $p < 0.001$) of the different functional groups and their interactions. (b) Mean observed basal soil respiration of soils containing a given class of family relative abundance. The linked points correspond to the different classes of increasing relative abundance (from left to right) for each family. The used symbols are the same as those used in (a). (c) Boxplots of the observed basal soil respiration sorted by assembly motifs, i.e., combinations of functional groups. The used symbols are the same as those used in (b). (d) Modeled versus observed basal soil respiration. Different symbols correspond to different assembly motifs, i.e., combinations of functional groups. The red line is the bisector. (b), (c) and (d) The dotted line is the mean observed basal soil respiration.

Jaillard, B.; Razanamalala, K.; Violle, C.; Bernard, L. Nonlinear Effects Induced by Interactions among Functional Groups of Bacteria and Fungi Regulate the Priming Effect in Malagasy Soils. *Microorganisms*.

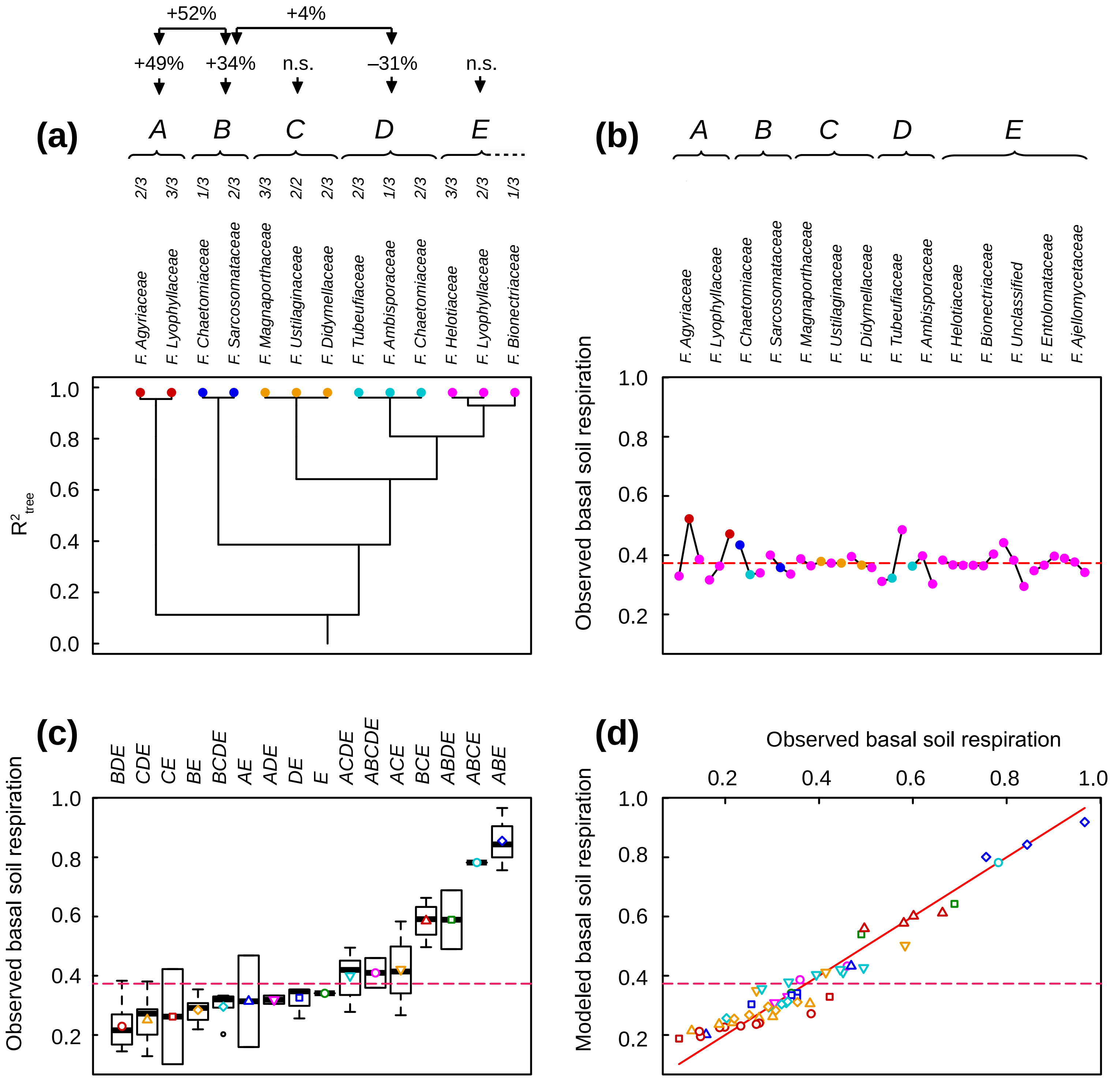

Figure S6. Clustering analysis of basal soil respiration after 42 days of incubation, explained by the relative abundance classes of fungal families in soil. (**a**) Clustering tree of classes of family relative abundance. The functional groups, and the classes of family relative abundance inside each functional group, are sorted from left to right by decreasing effect on the basal soil respiration. Each functional group has a color attributed: red for A, dark blue for B, yellow for C, light blue for D, and pink for E. The above box reports the significant effects (at $p < 0.001$) of the different functional groups and their interactions. (**b**) Mean observed basal soil respiration of soils containing a given class of family relative abundance. The linked points correspond to the different classes of increasing relative abundance (from left to right) for each family. The used symbols are the same as those used in (**a**). (**c**) Boxplots of the observed basal soil respiration sorted by assembly motifs, i.e., combinations of functional groups. The used symbols are the same as those used in (**b**). (**d**) Modeled versus observed soil property. Different symbols correspond to different assembly motifs, i.e., combinations of functional groups. The red line is the bisector. (**b**), (**c**) and (**d**) The dotted line is the mean observed basal soil respiration.

Jaillard, B.; Razanamalala, K.; Violle, C.; Bernard, L. Nonlinear Effects Induced by Interactions among Functional Groups of Bacteria and Fungi Regulate the Priming Effect in Malagasy Soils. *Microorganisms*.

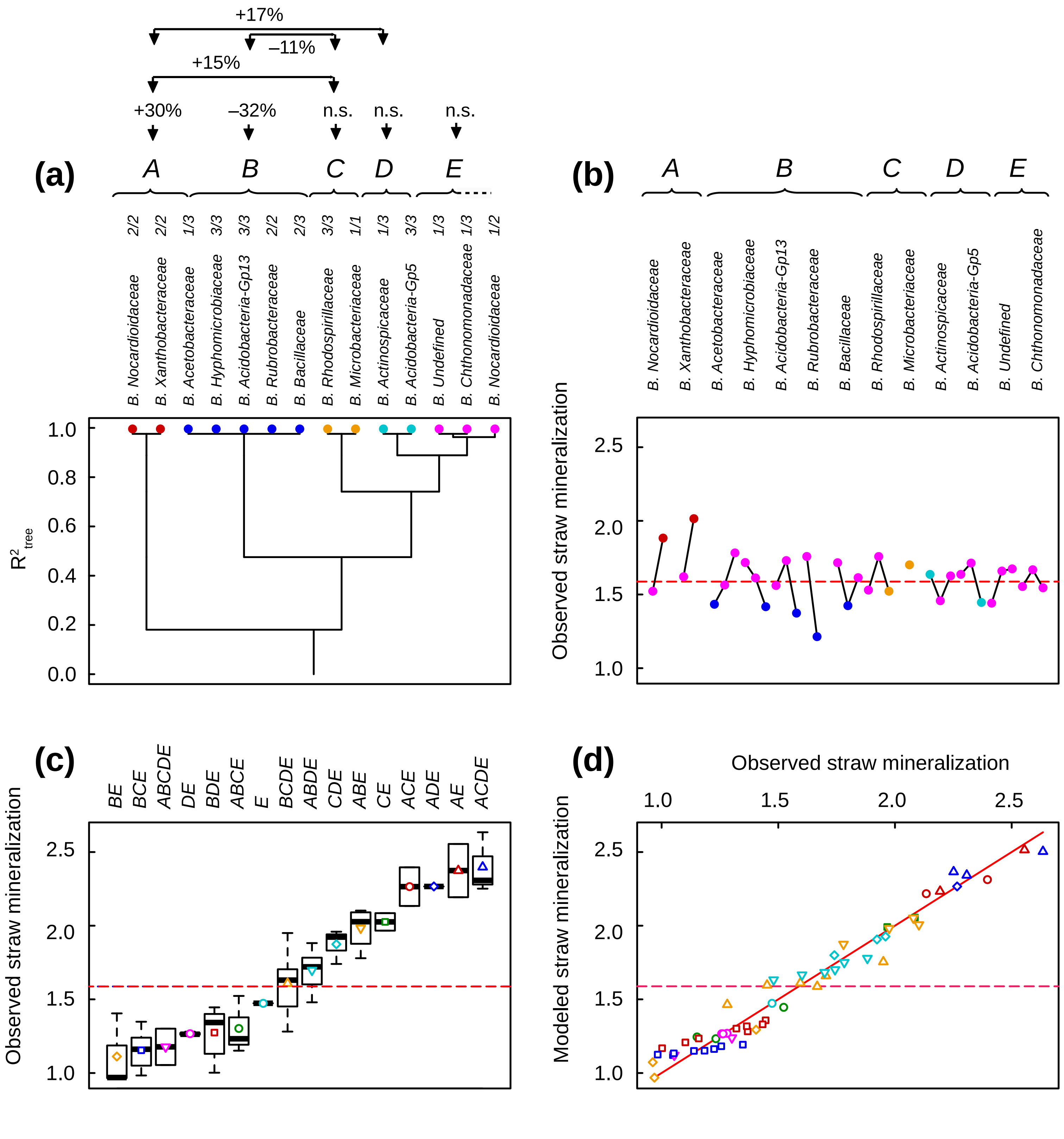

Figure S7. Clustering analysis of straw mineralization after seven days of incubation, explained by the relative abundance classes of bacterial families in soil. (**a**) Clustering tree of classes of family relative abundance. The functional groups, and the classes of family relative abundance inside each functional group, are sorted from left to right by decreasing effect on the straw mineralization. Each functional group has a color attributed: red for A, dark blue for B, yellow for C, light blue for D, and pink for E. The above box reports the significant effects (at $p < 0.001$) of the different functional groups and their interactions. (**b**) Mean observed straw mineralization of soils containing a given class of family relative abundance. The linked points correspond to the different classes of increasing relative abundance (from left to right) for each family. The used symbols are the same as those used in (**a**). (**c**) Boxplots of the observed straw mineralization sorted by assembly motifs, i.e., combinations of functional groups. The used symbols are the same as those used in (**b**). (**d**) Modeled versus observed straw mineralization. Different symbols correspond to different assembly motifs, i.e., combinations of functional groups. The red line is the bisector. (**b**), (**c**) and (**d**) The dotted line is the mean observed straw mineralization.

Jaillard, B.; Razanamalala, K.; Violle, C.; Bernard, L. Nonlinear Effects Induced by Interactions among Functional Groups of Bacteria and Fungi Regulate the Priming Effect in Malagasy Soils. *Microorganisms.*

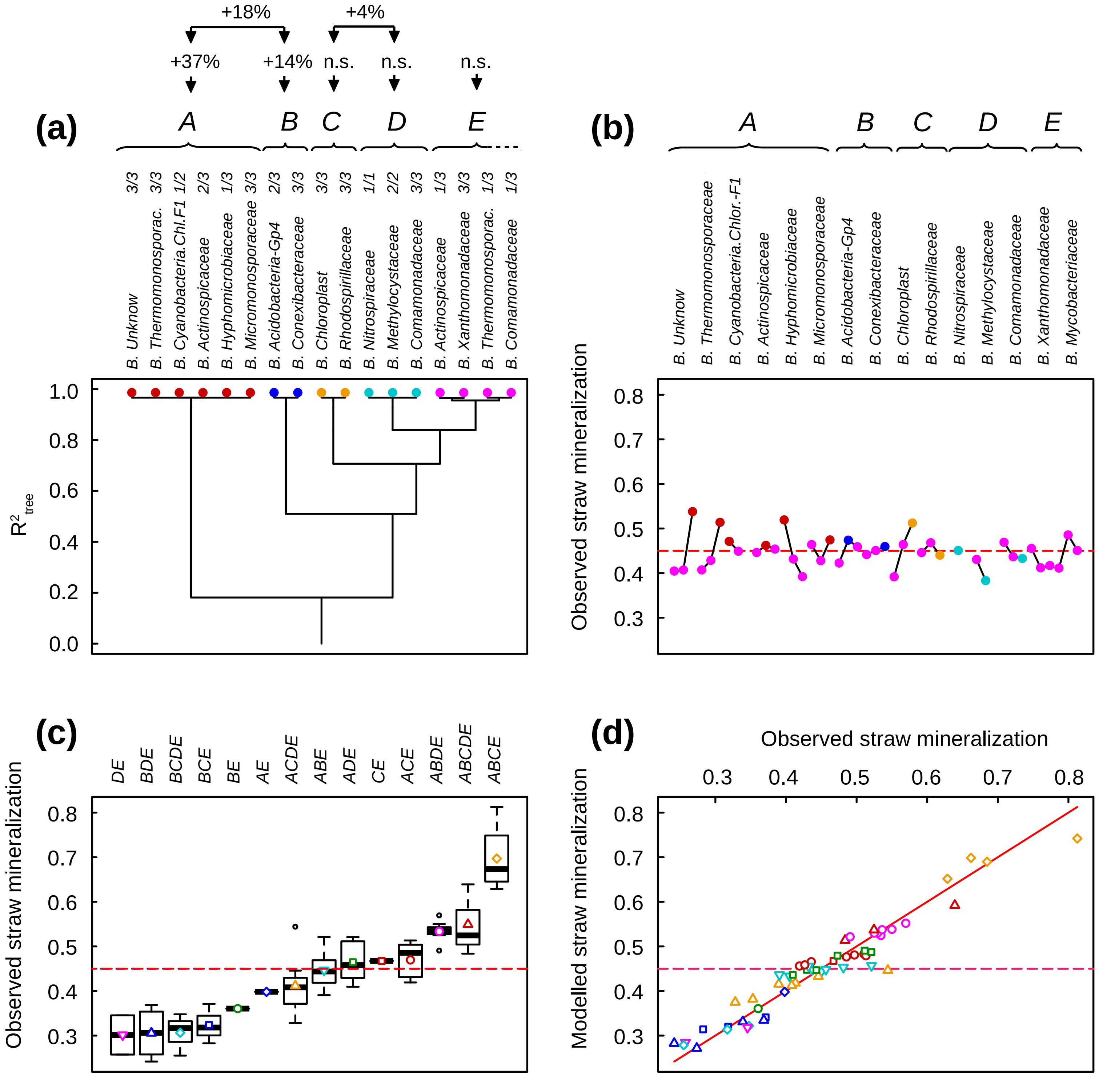

Figure S8. Clustering analysis of straw mineralization after 42 days of incubation, explained by the relative abundance classes of bacterial families in soil. (**a**) Clustering tree of classes of family relative abundance. The functional groups, and the classes of family relative abundance inside each functional group, are sorted from left to right by decreasing effect on the straw mineralization. Each functional group has a color attributed: red for A, dark blue for B, yellow for C, light blue for D, and pink for E. The above box reports the significant effects (at $p < 0.001$) of the different functional groups and their interactions. (**b**) Mean observed straw mineralization of soils containing a given class of family relative abundance. The linked points correspond to the different classes of increasing relative abundance (from left to right) for each family. The used symbols are the same as those used in (**a**). (**c**) Boxplots of the observed straw mineralization sorted by assembly motifs, i.e., combinations of functional groups. The used symbols are the same as those used in (**b**). (**d**) Modeled versus observed straw mineralization. Different symbols correspond to different assembly motifs, i.e., combinations of functional groups. The red line is the bisector. (**b**), (**c**) and (**d**) The dotted line is the mean observed straw mineralization.

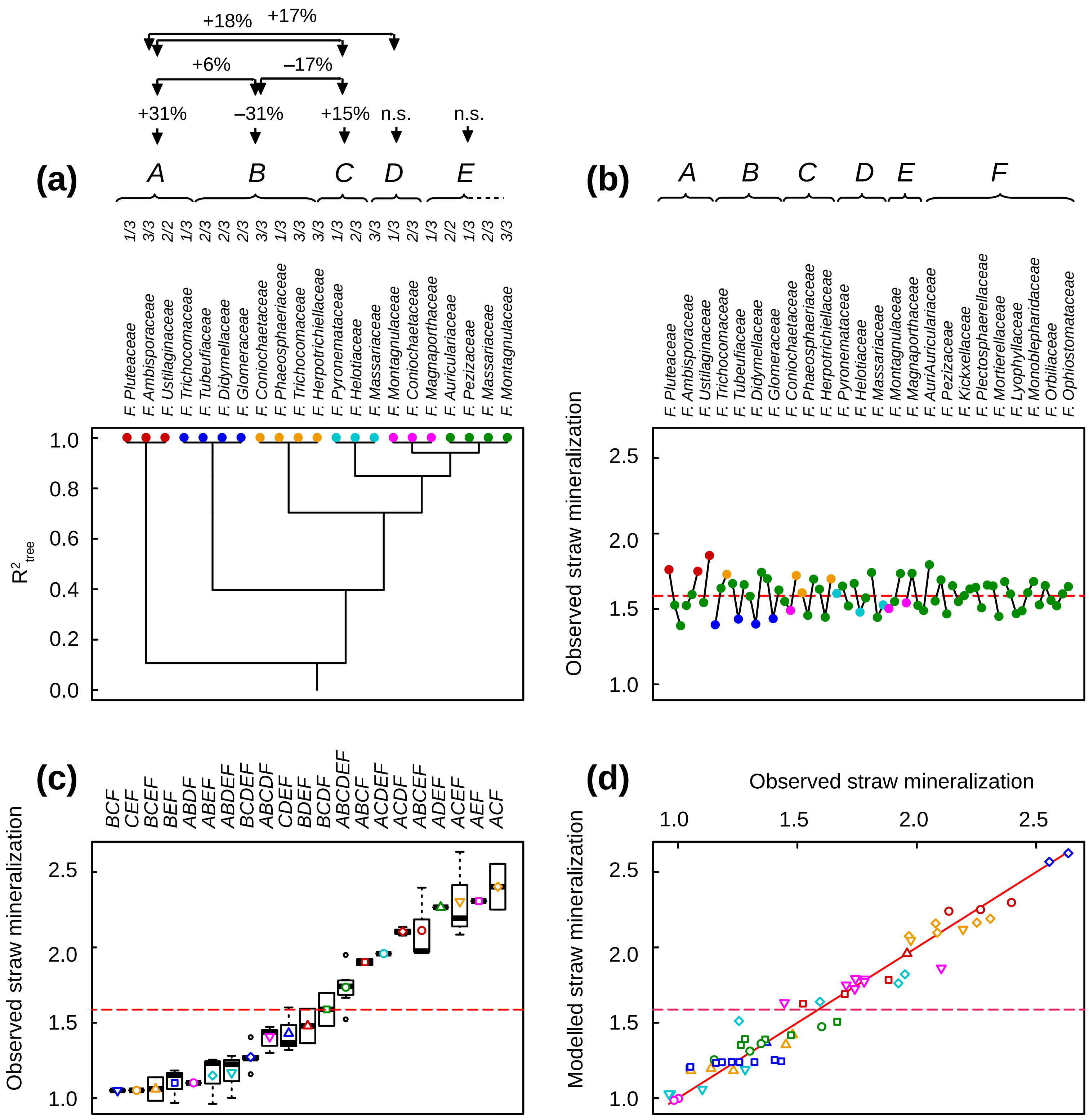

Figure S9. Clustering analysis of straw mineralization after seven days of incubation, explained by the relative abundance classes of fungal families in soil. (**a**) Clustering tree of classes of family relative abundance. The functional groups, and the classes of family relative abundance inside each functional group, are sorted from left to right by decreasing effect on the straw mineralization. Each functional group has a color attributed: red for A, dark blue for B, yellow for C, light blue for D, pink for E, and green for F. The above box reports the significant effects (at $p < 0.001$) of the different functional groups and their interactions. (b) Mean observed straw mineralization of soils containing a given class of family relative abundance. The linked points correspond to the different classes of increasing relative abundance (from left to right) for each family. The used symbols are the same as those used in (**a**). (**c**) Boxplots of the observed straw mineralization sorted by assembly motifs, i.e., combinations of functional groups. The used symbols are the same as those used in (**b**). (**d**) Modeled versus observed straw mineralization. Different symbols correspond to different assembly motifs, i.e., combinations of functional groups. The red line is the bisector. (**b**), (**c**) and (**d**) The dotted line is the mean observed straw mineralization.

Jaillard, B.; Razanamalala, K.; Violle, C.; Bernard, L. Nonlinear Effects Induced by Interactions among Functional Groups of Bacteria and Fungi Regulate the Priming Effect in Malagasy Soils. *Microorganisms.*

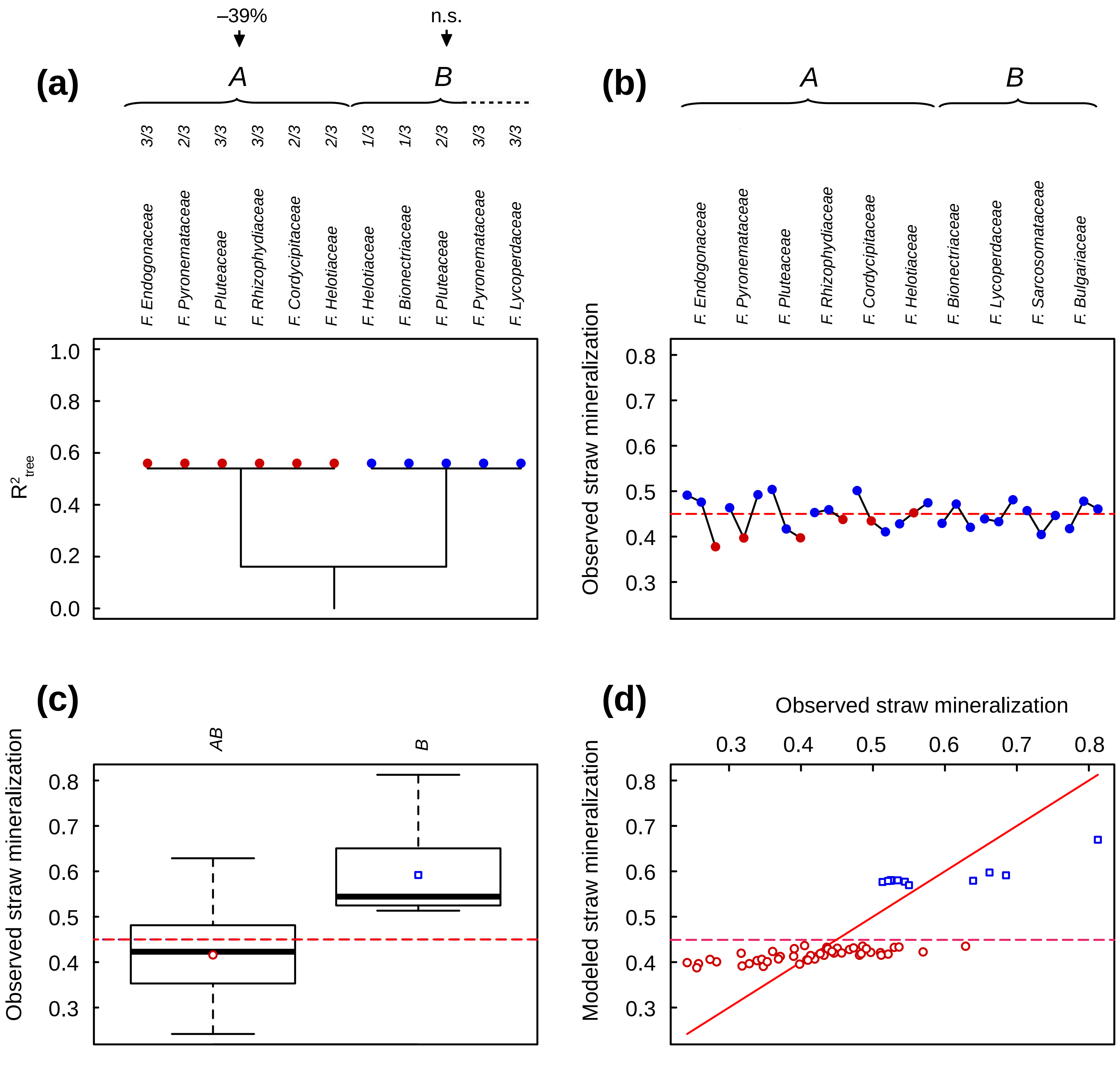

Figure S10. Clustering analysis of straw mineralization after 42 days of incubation, explained by the relative abundance classes of fungal families in soil. (**a**) Clustering tree of classes of family relative abundance. The functional groups, and the classes of family relative abundance inside each functional group, are sorted from left to right by decreasing effect on the straw mineralization. Each functional group has a color attributed: red for A, and dark blue for B. The above box reports the significant effects (at p < 0.001) of the different functional groups and their interactions. (**b**) Mean observed straw mineralization of soils containing a given class of family relative abundance. The linked points correspond to the different classes of increasing relative abundance (from left to right) for each family. The used symbols are the same as those used in (**a**). (**c**) Boxplots of the observed straw mineralization sorted by assembly motifs, i.e., combinations of functional groups. The used symbols are the same as those used in (**b**). (**d**) Modeled versus observed straw mineralization. Different symbols correspond to different assembly motifs, i.e., combinations of functional groups. The red line is the bisector. (**b**), (**c**) and (**d**) The dotted line is the mean observed straw mineralization.

Jaillard, B.; Razanamalala, K.; Violle, C.; Bernard, L. Nonlinear Effects Induced by Interactions among Functional Groups of Bacteria and Fungi Regulate the Priming Effect in Malagasy Soils. *Microorganisms.*

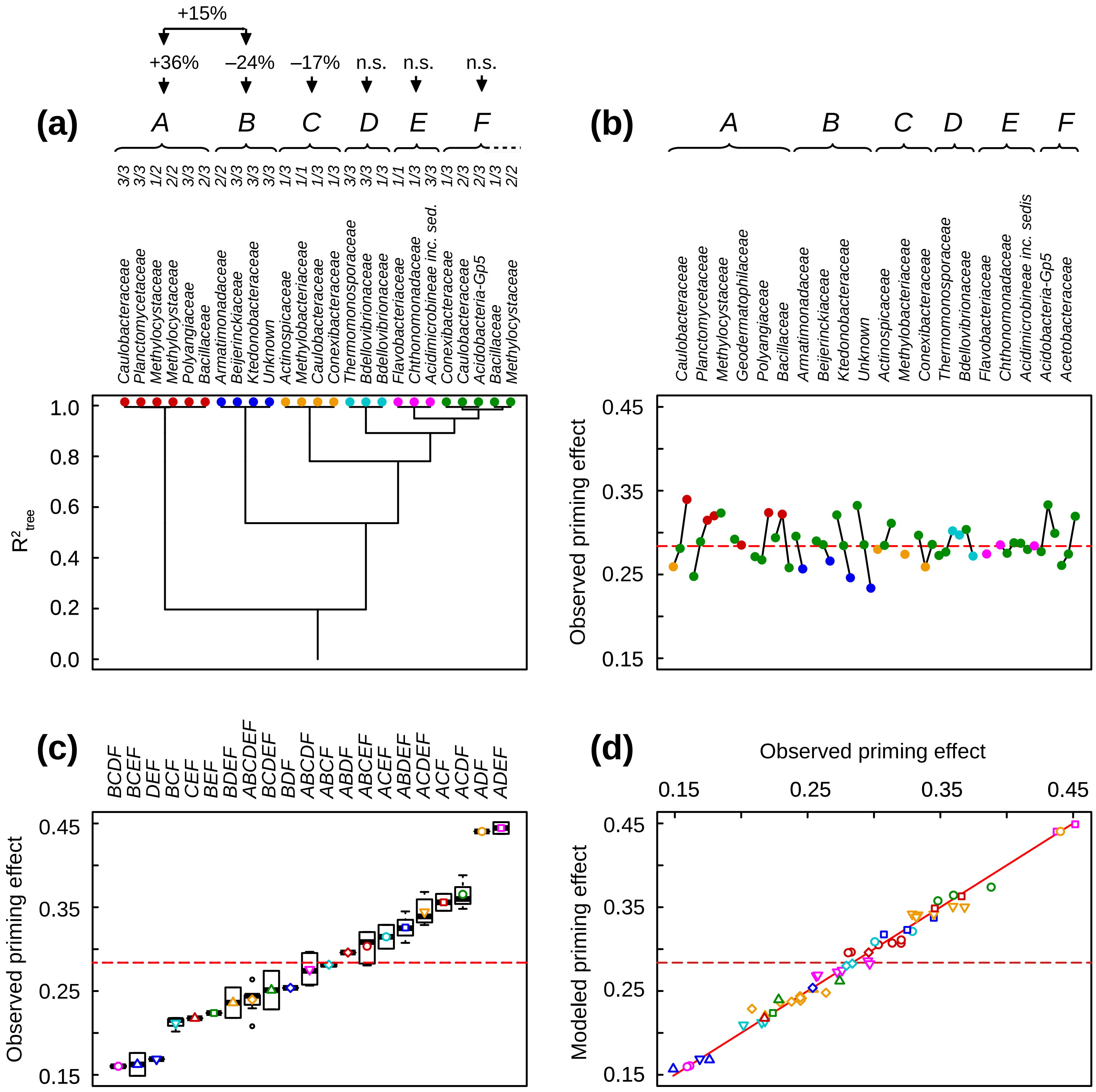

Figure S11. Clustering analysis of the priming effect after seven days of incubation, explained by the relative abundance classes of bacterial families in soil. (**a**) Clustering tree of classes of family relative abundance. The functional groups, and the classes of family relative abundance inside each functional group, are sorted from left to right by decreasing effect on the priming effect. Each functional group has a color attributed: red for A, dark blue for B, yellow for C, light blue for D, pink for E, and green for F. The above box reports the significant effects (at p < 0.001) of the different functional groups and their interactions. (**b**) Mean observed priming effect of soils containing a given class of family relative abundance. The linked points correspond to the different classes of increasing relative abundance (from left to right) for each family. The used symbols are the same as those used in (**a**). (**c**) Boxplots of the observed priming effect sorted by assembly motifs, i.e., combinations of functional groups. The used symbols are the same as those used in (**b**). (**d**) Modeled versus observed priming effect. Different symbols correspond to different assembly motifs, i.e., combinations of functional groups. The red line is the bisector. (**b**), (**c**) and (**d**) The dotted line is the mean observed priming effect.

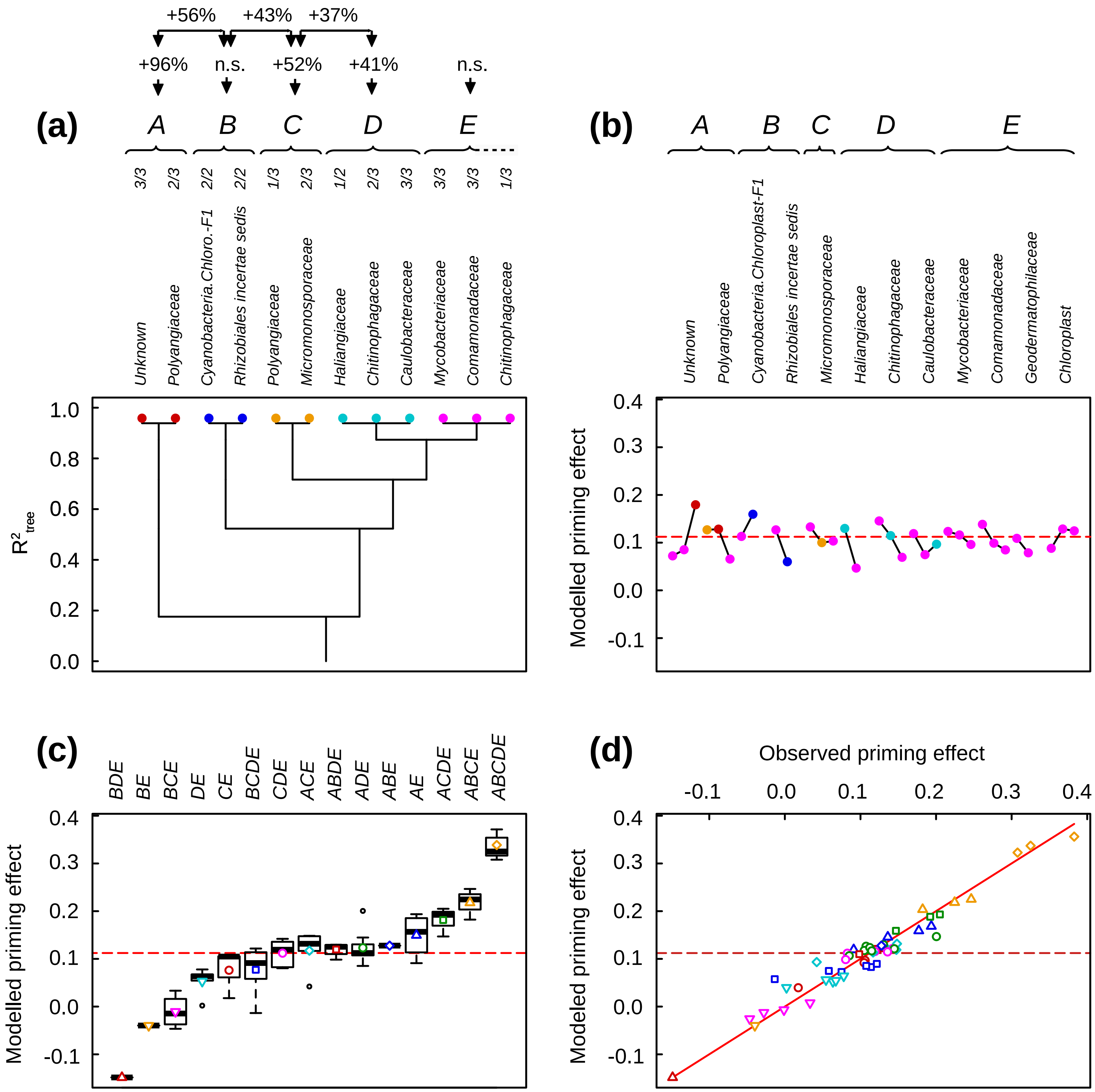

Figure S12. Clustering analysis of the priming effect after 42 days of incubation, explained by the relative abundance classes of bacterial families in soil. (**a**) Clustering tree of classes of family relative abundance. The functional groups, and the classes of family relative abundance inside each functional group, are sorted from left to right by decreasing effect on the priming effect. Each functional group has a color attributed: red for A, dark blue for B, yellow for C, light blue for D, and pink for E. The above box reports the significant effects (at $p < 0.001$) of the different functional groups and their interactions. (**b**) Mean observed priming effect of soils containing a given class of family relative abundance. The linked points correspond to the different classes of increasing relative abundance (from left to right) for each family. The used symbols are the same as those used in (**a**). (**c**) Boxplots of the observed priming effect sorted by assembly motifs, i.e., combinations of functional groups. The used symbols are the same as those used in (**b**). (**d**) Modeled versus observed priming effect. Different symbols correspond to different assembly motifs, i.e., combinations of functional groups. The red line is the bisector. (**b**), (**c**) and (**d**) The dotted line is the mean observed priming effect.

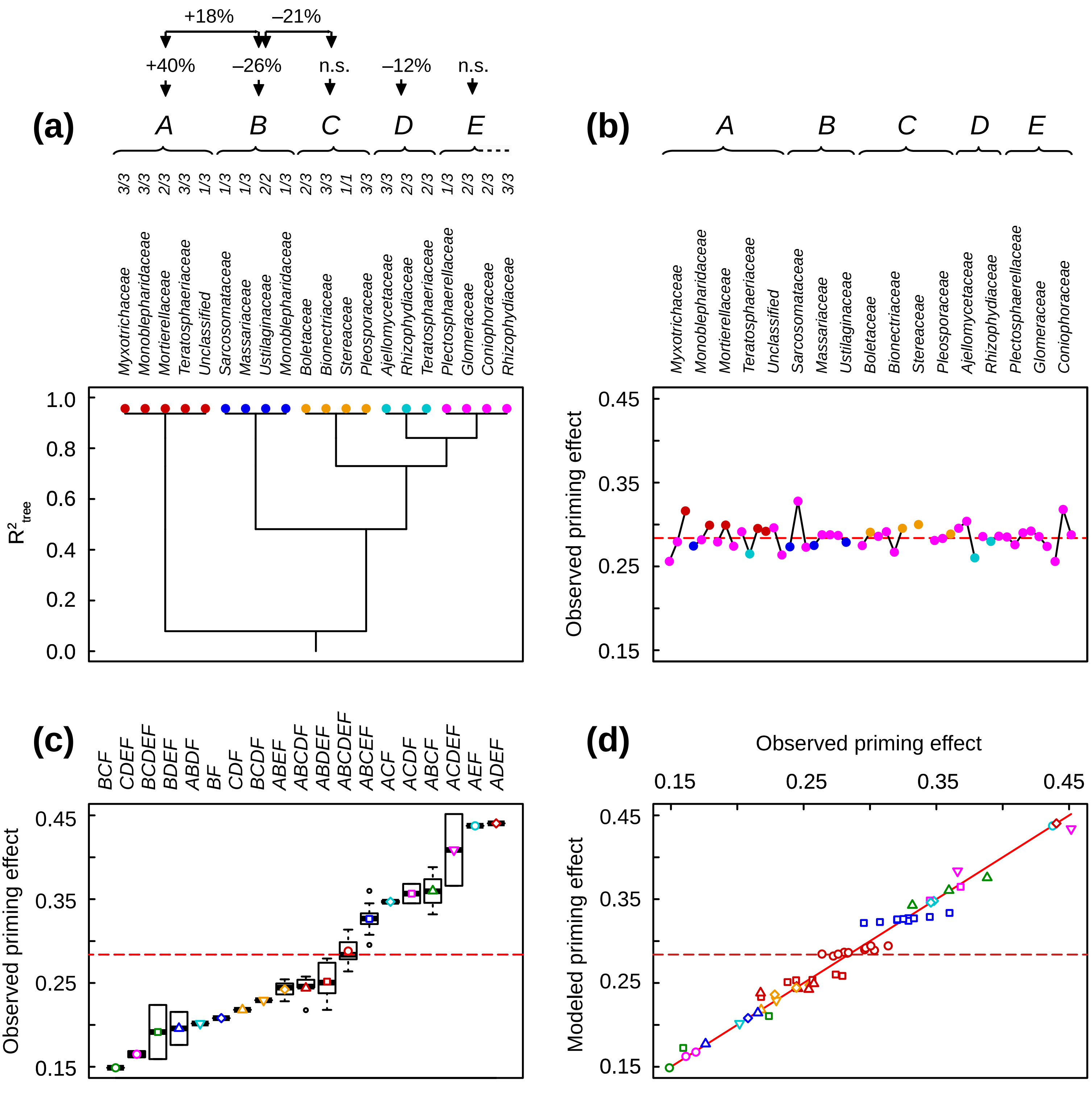

Figure S13. Clustering analysis of the priming effect after seven days of incubation, explained by the relative abundance classes of fungal families in soil. (**a**) Clustering tree of classes of family relative abundance. The functional groups, and the classes of family relative abundance inside each functional group, are sorted from left to right by decreasing effect on the priming effect. Each functional group has a color attributed: red for A, dark blue for B, yellow for C, light blue for D, and pink for E. The above box reports the significant effects (at $p < 0.001$) of the different functional groups and their interactions. (**b**) Mean observed priming effect of soils containing a given class of family relative abundance. The linked points correspond to the different classes of increasing relative abundance (from left to right) for each family. The used symbols are the same as those used in (**a**). (**c**) Boxplots of the observed priming effect sorted by assembly motifs, i.e., combinations of functional groups. The used symbols are the same as those used in (**b**). (**d**) Modeled versus observed priming effect. Different symbols correspond to different assembly motifs, i.e., combinations of functional groups. The red line is the bisector. (**b**), (**c**) and (**d**) The dotted line is the mean observed priming effect.

Jaillard, B.; Razanamalala, K.; Violle, C.; Bernard, L. Nonlinear Effects Induced by Interactions among Functional Groups of Bacteria and Fungi Regulate the Priming Effect in Malagasy Soils. ***Microorganisms.***

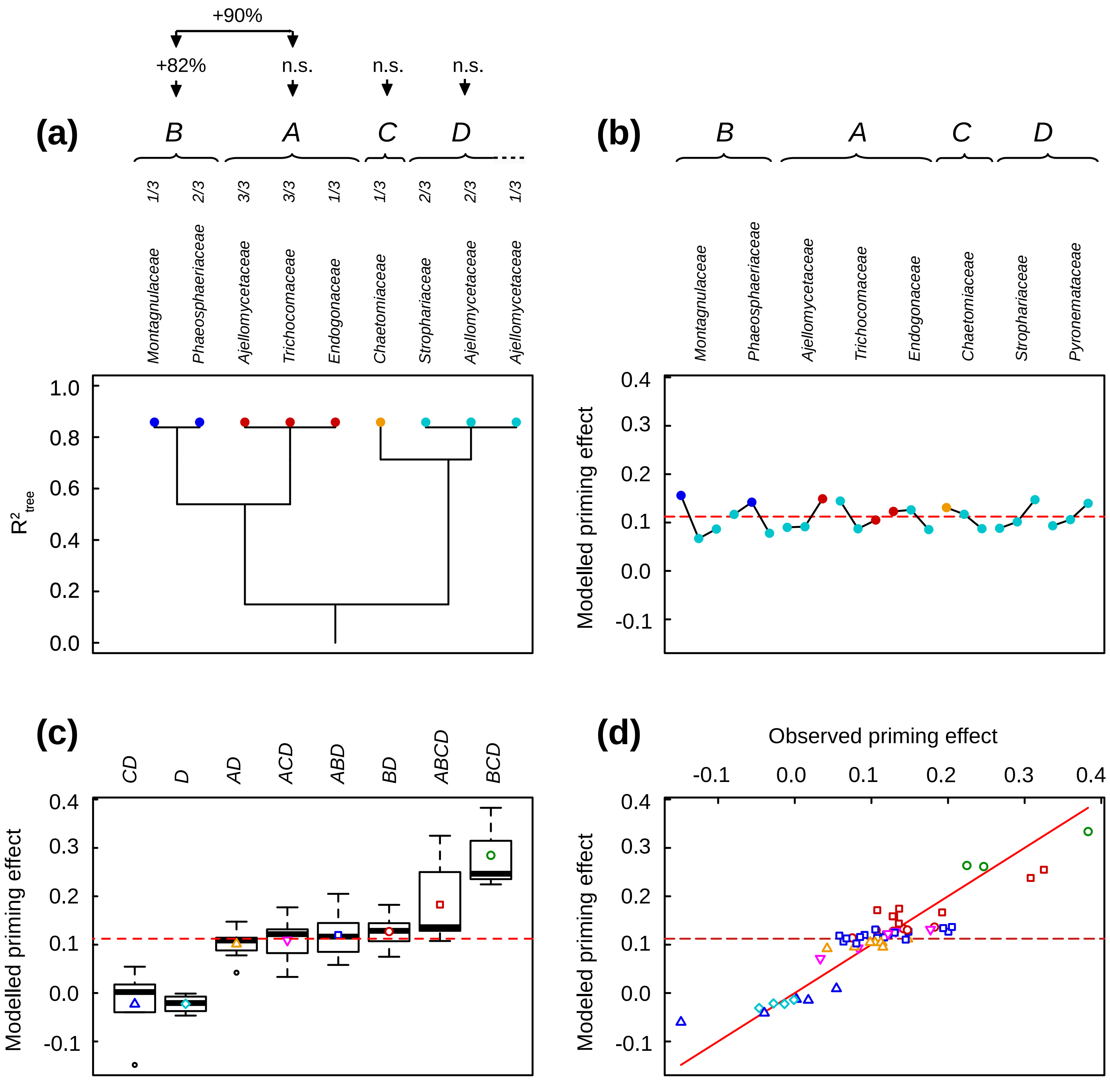

Figure S14. Clustering analysis of the priming effect after 42 days of incubation, explained by the relative abundance classes of fungal families in soil. (**a**) Clustering tree of classes of family relative abundance. The functional groups, and the classes of family relative abundance inside each functional group, are sorted from left to right by decreasing effect on the priming effect. Each functional group has a color attributed: red for A, dark blue for B, yellow for C, and light blue for D. The above box reports the significant effects (at $p < 0.001$) of the different functional groups and their interactions. (**b**) Mean observed priming effect of soils containing a given class of family relative abundance. The linked points correspond to the different classes of increasing relative abundance (from left to right) for each family. The used symbols are the same as those used in (**a**). (**c**) Boxplots of the observed priming effect sorted by assembly motifs, i.e., combinations of functional groups. The used symbols are the same as those used in (**b**). (**d**) Modeled versus observed priming effect. Different symbols correspond to different assembly motifs, i.e., combinations of functional groups. The red line is the bisector. (**b**), (**c**) and (**d**) The dotted line is the mean observed priming effect.

Jaillard, B.; Razanamalala, K.; Violle, C.; Bernard, L. Nonlinear Effects Induced by Interactions among Functional Groups of Bacteria and Fungi Regulate the Priming Effect in Malagasy Soils. *Microorganisms.*

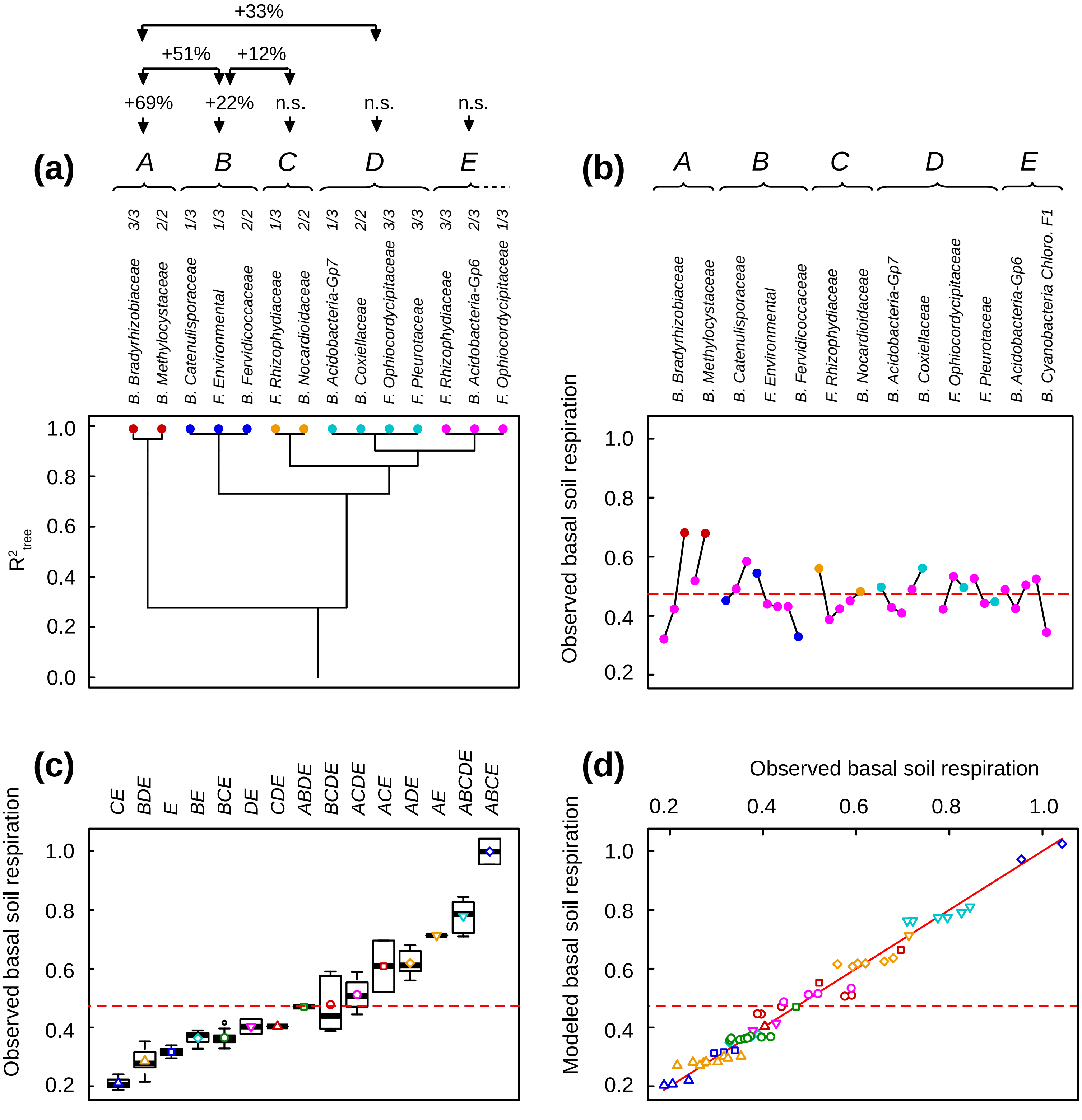

Figure S15. Clustering analysis of basal soil respiration after seven days of incubation, explained by the relative abundance classes of bacterial and fungal families together in soil. (**a**) Clustering tree of classes of family relative abundance. The functional groups, and the classes of family relative abundance inside each functional group, are sorted from left to right by decreasing effect on the basal soil respiration. Each functional group has a color attributed: red for A, dark blue for B, yellow for C, light blue for D, and pink for E. The above box reports the significant effects (at $p < 0.001$) of the different functional groups and their interactions. (**b**) Mean observed basal soil respiration of soils containing a given class of family relative abundance. The linked points correspond to the different classes of increasing relative abundance (from left to right) for each family. The used symbols are the same as those used in (**a**). (**c**) Boxplots of the observed basal soil respiration sorted by assembly motifs, i.e., combinations of functional groups. The used symbols are the same as those used in (**b**). (**d**) Modeled versus observed basal soil respiration. Different symbols correspond to different assembly motifs, i.e., combinations of functional groups. The red line is the bisector. (**b**), (**c**) and (**d**) The dotted line is the mean observed basal soil respiration.

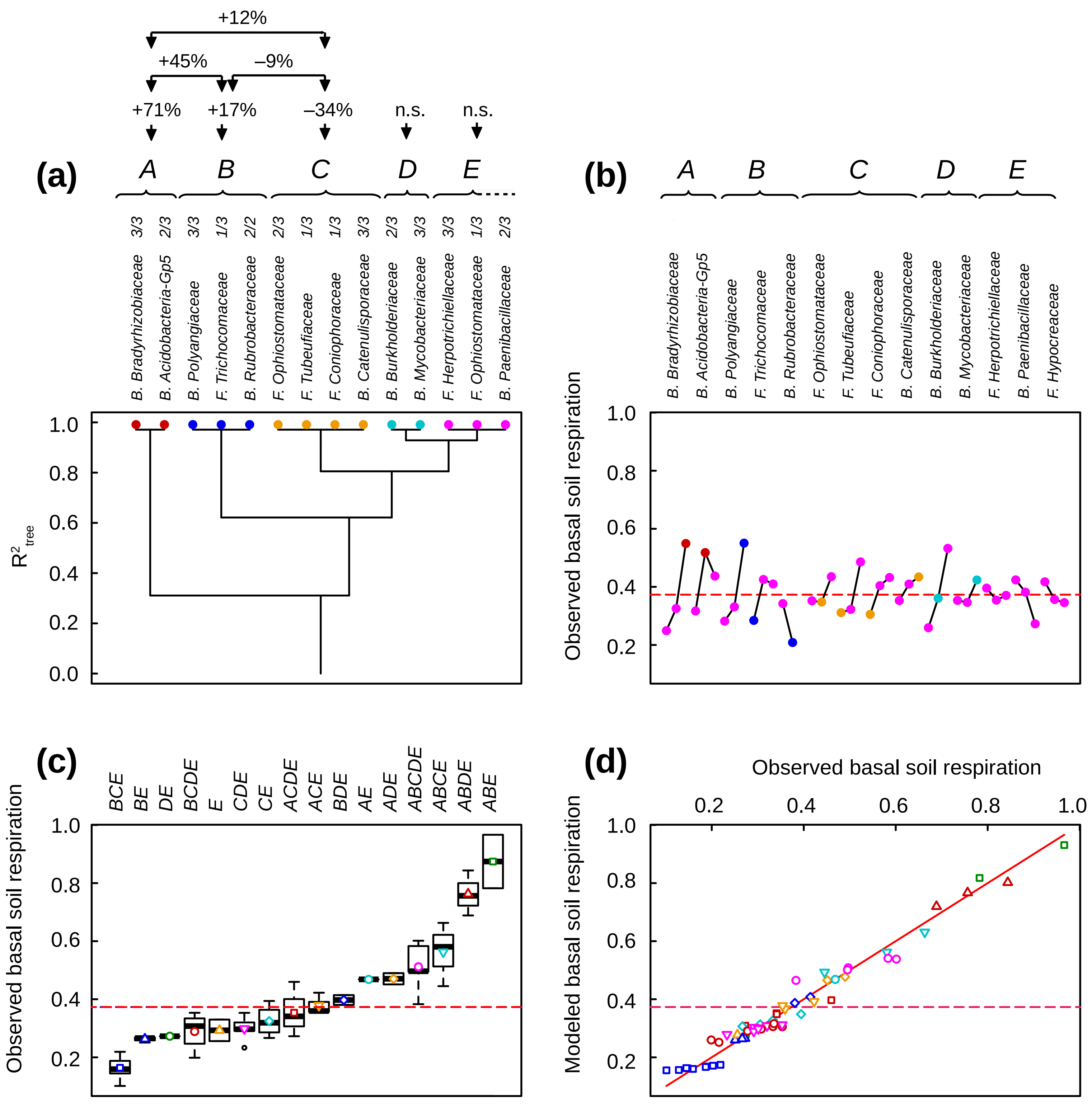

Figure S16. Clustering analysis of basal soil respiration after 42 days of incubation, explained by the relative abundance classes of bacterial and fungal families together in soil. (**a**) Clustering tree of classes of family relative abundance. The functional groups, and the classes of family relative abundance inside each functional group, are sorted from left to right by decreasing effect on the basal soil respiration. Each functional group has a color attributed: red for A, dark blue for B, yellow for C, light blue for D, and pink for E. The above box reports the significant effects (at $p < 0.001$) of the different functional groups and their interactions. (**b**) Mean observed property of soils containing a given class of family relative abundance. The linked points correspond to the different classes of increasing relative abundance (from left to right) for each family. The used symbols are the same as those used in (**a**). (**c**) Boxplots of the observed basal soil respiration sorted by assembly motifs, i.e., combinations of functional groups. The used symbols are the same as those used in (**b**). (**d**) Modeled versus observed basal soil respiration. Different symbols correspond to different assembly motifs, i.e., combinations of functional groups. The red line is the bisector. (**b**), (**c**) and (**d**) The dotted line is the mean observed basal soil respiration.

Jaillard, B.; Razanamalala, K.; Violle, C.; Bernard, L. Nonlinear Effects Induced by Interactions among Functional Groups of Bacteria and Fungi Regulate the Priming Effect in Malagasy Soils. *Microorganisms.*

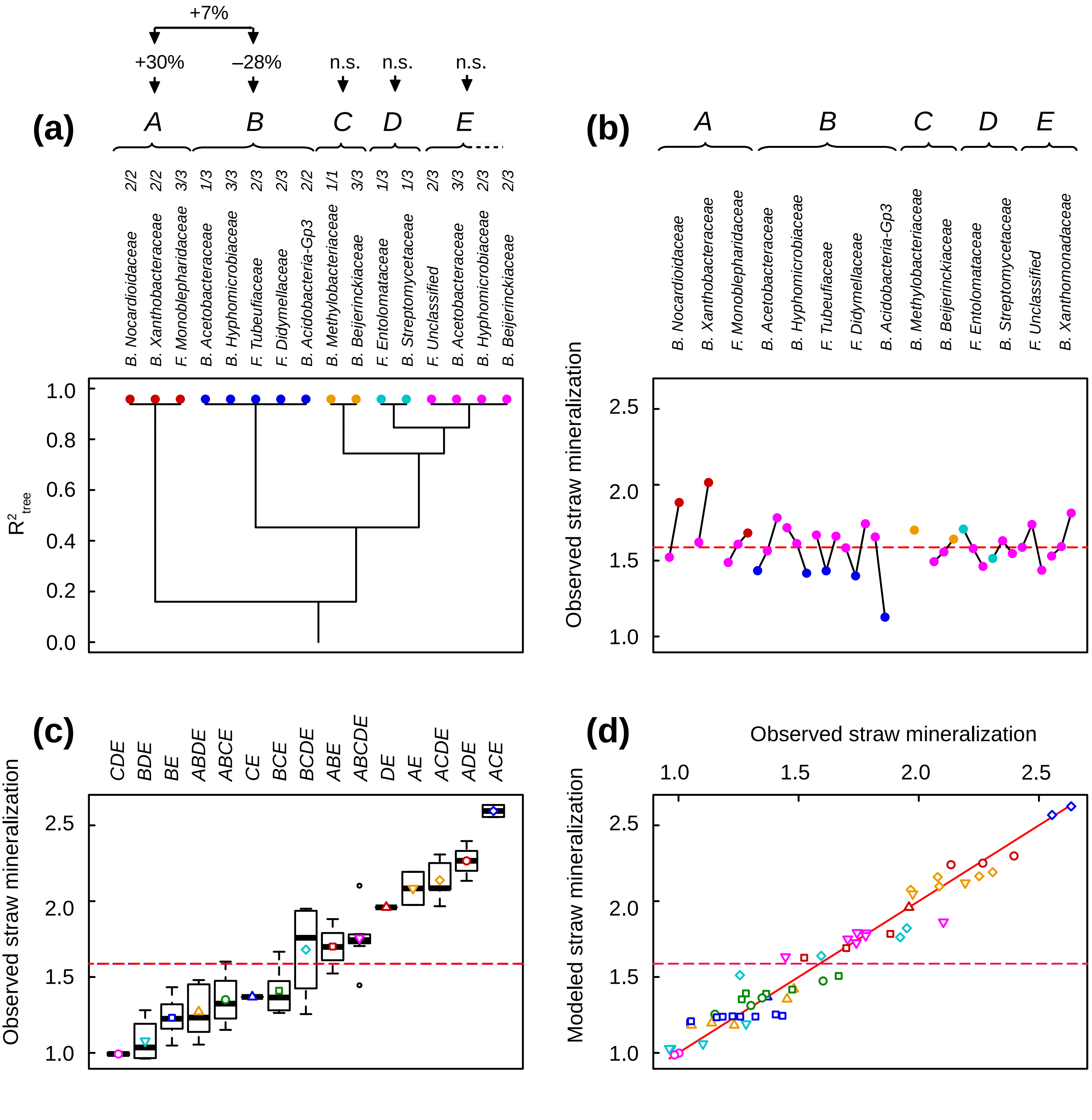

Figure S17. Clustering analysis of straw mineralization after seven days of incubation, explained by the relative abundance classes of bacterial and fungal families together in soil. (**a**) Clustering tree of classes of family relative abundance. The functional groups, and the classes of family relative abundance inside each functional group, are sorted from left to right by decreasing effect on the straw mineralization. Each functional group has a color attributed: red for A, dark blue for B, yellow for C, light blue for D, and pink for E. The above box reports the significant effects (at $p < 0.001$) of the different functional groups and their interactions. (**b**) Mean observed straw mineralization of soils containing a given class of family relative abundance. The linked points correspond to the different classes of increasing relative abundance (from left to right) for each family. The used symbols are the same as those used in (**a**). (**c**) Boxplots of the observed straw mineralization sorted by assembly motifs, i.e., combinations of functional groups. The used symbols are the same as those used in (**b**). (**d**) Modeled versus observed straw mineralization. Different symbols correspond to different assembly motifs, i.e., combinations of functional groups. The red line is the bisector. (**b**), (**c**) and (**d**) The dotted line is the mean observed straw mineralization.

Jaillard, B.; Razanamalala, K.; Violle, C.; Bernard, L. Nonlinear Effects Induced by Interactions among Functional Groups of Bacteria and Fungi Regulate the Priming Effect in Malagasy Soils. *Microorganisms.*

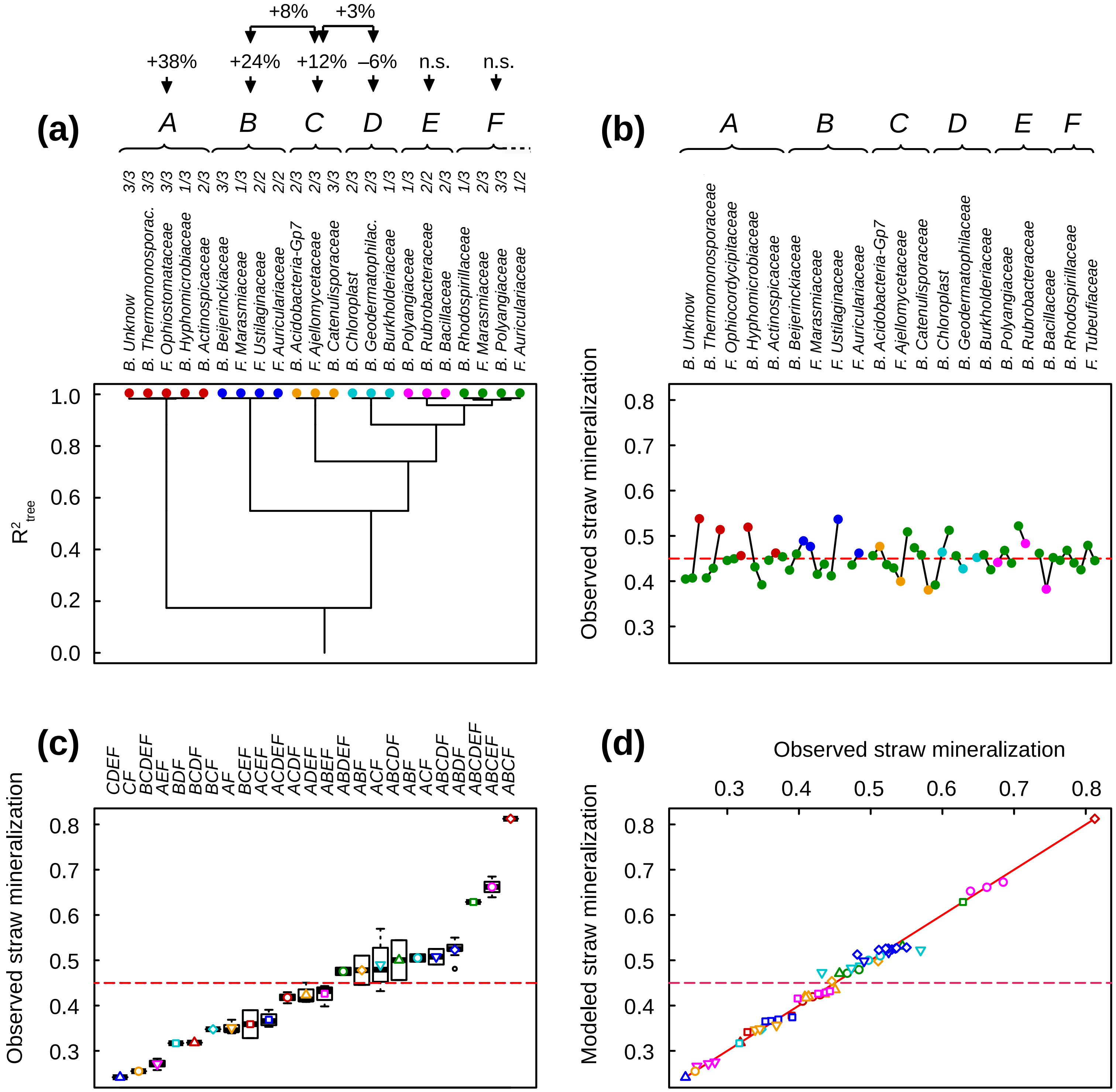

Figure S18. Clustering analysis of straw mineralization after 42 days of incubation, explained by the relative abundance classes of bacterial and fungal families together in soil. (**a**) Clustering tree of classes of family relative abundance. The functional groups, and the classes of family relative abundance inside each functional group, are sorted from left to right by decreasing effect on the straw mineralization. Each functional group has a color attributed: red for A, dark blue for B, yellow for C, light blue for D, pink for E, and green for F. The above box reports the significant effects (at $p < 0.001$) of the different functional groups and their interactions. (**b**) Mean observed straw mineralization of soils containing a given class of family relative abundance. The linked points correspond to the different classes of increasing relative abundance (from left to right) for each family. The used symbols are the same as those used in (**a**). (**c**) Boxplots of the observed straw mineralization sorted by assembly motifs, i.e., combinations of functional groups. The used symbols are the same as those used in (**b**). (**d**) Modeled versus observed straw mineralization. Different symbols correspond to different assembly motifs, i.e., combinations of functional groups. The red line is the bisector. (**b**), (**c**) and (**d**) The dotted line is the mean observed straw mineralization.

Jaillard, B.; Razanamalala, K.; Violle, C.; Bernard, L. Nonlinear Effects Induced by Interactions among Functional Groups of Bacteria and Fungi Regulate the Priming Effect in Malagasy Soils. ***Microorganisms.***

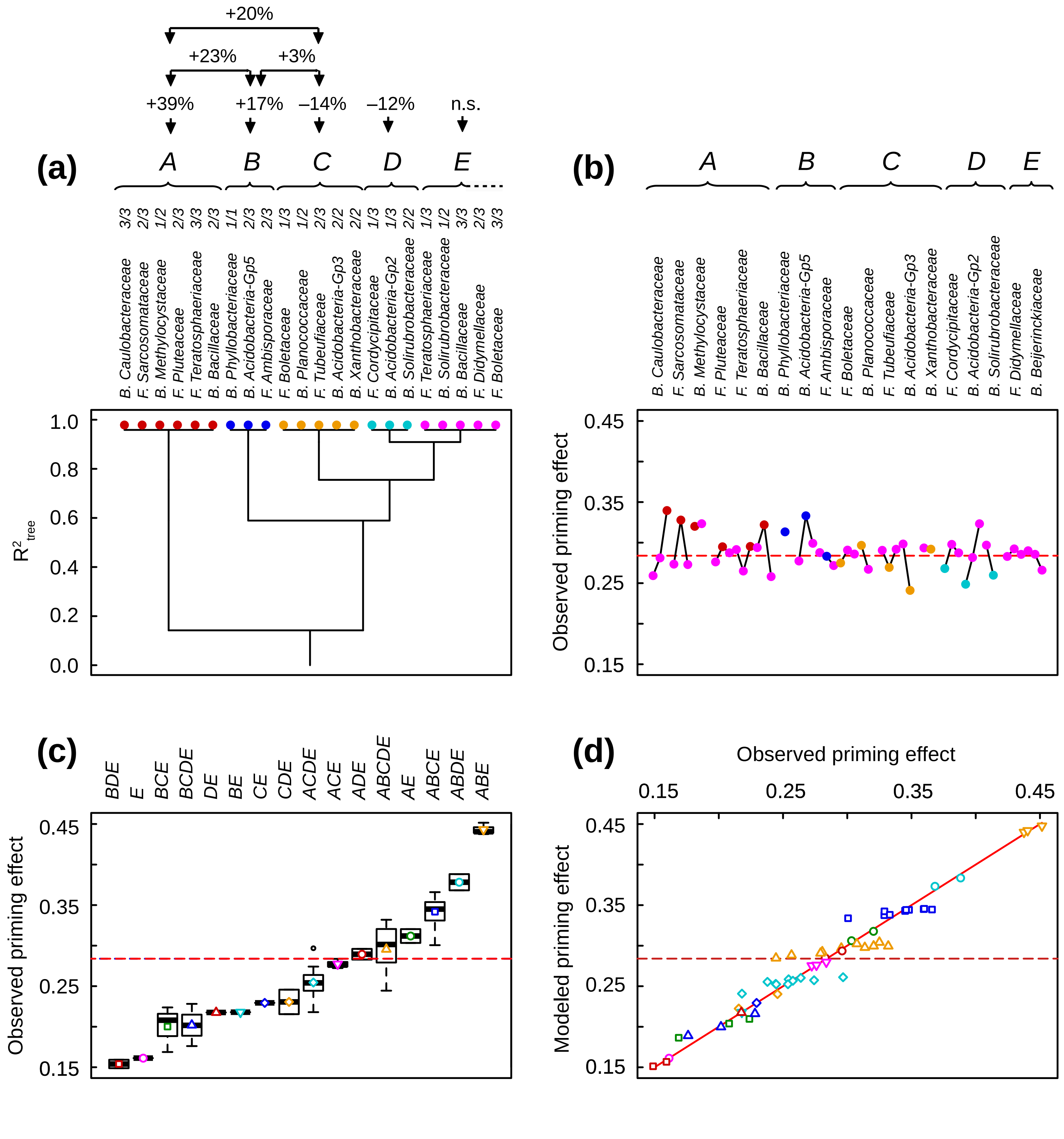

Figure S19. Clustering analysis of the priming effect after seven days of incubation, explained by the relative abundance classes of bacterial and fungal families together in soil. (**a**) Clustering tree of classes of family relative abundance. The functional groups, and the classes of family relative abundance inside each functional group, are sorted from left to right by decreasing effect on the priming effect. Each functional group has a color attributed: red for A, dark blue for B, yellow for C, light blue for D, and pink for E. The above box reports the significant effects (at $p < 0.001$) of the different functional groups and their interactions. (**b**) Mean observed priming effect of soils containing a given class of family relative abundance. The linked points correspond to the different classes of increasing relative abundance (from left to right) for each family. The used symbols are the same as those used in (**a**). (**c**) Boxplots of the observed priming effect sorted by assembly motifs, i.e., combinations of functional groups. The used symbols are the same as those used in (**b**). (**d**) Modeled versus observed priming effect. Different symbols correspond to different assembly motifs, i.e., combinations of functional groups. The red line is the bisector. (**b**), (**c**) and (**d**) The dotted line is the mean observed priming effect.

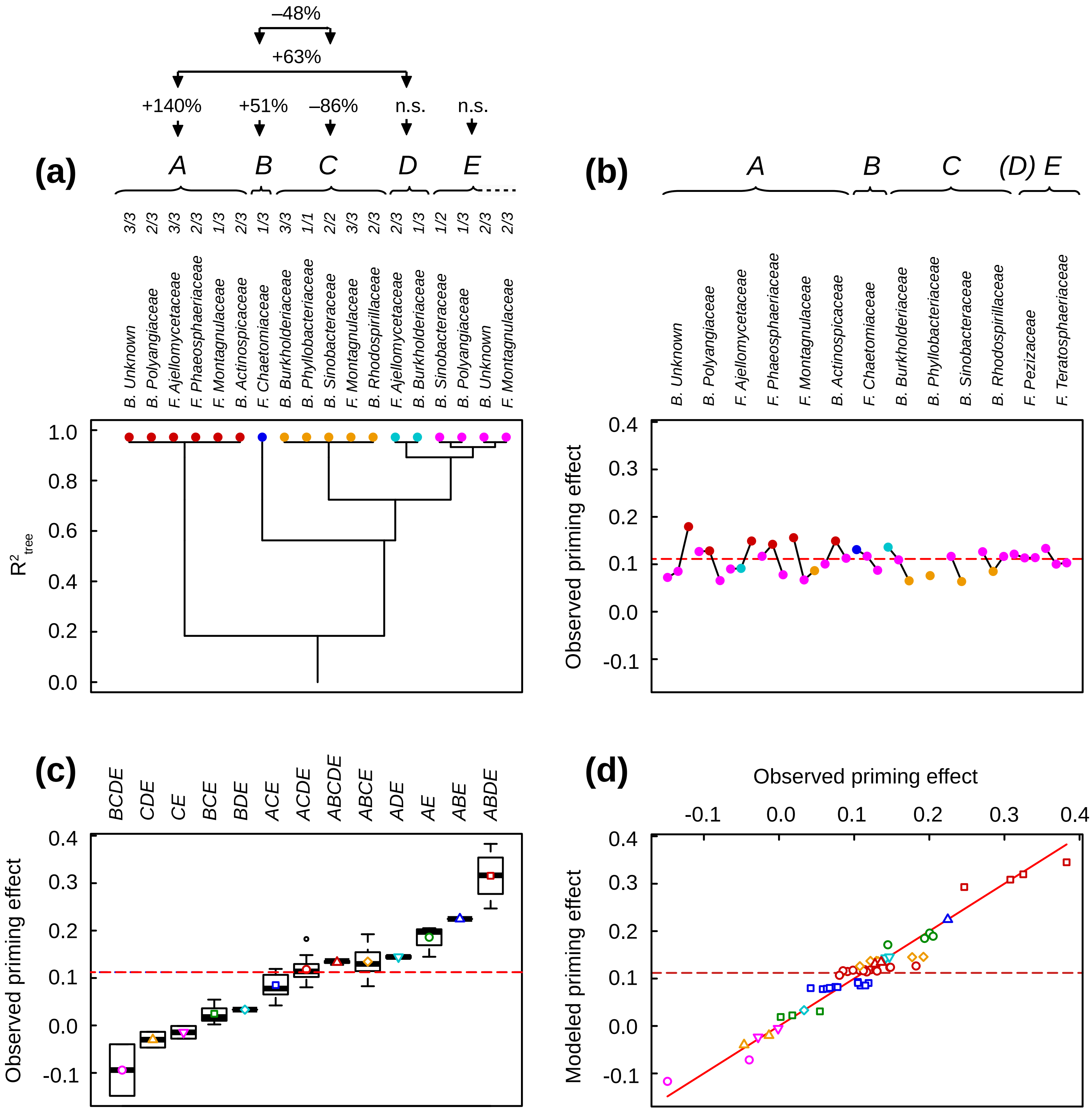

Figure S20. Clustering analysis of the priming effect after 42 days of incubation, explained by the relative abundance classes of bacterial and fungal families together in soil. (**a**) Clustering tree of classes of family relative abundance. The functional groups, and the classes of family relative abundance inside each functional group, are sorted from left to right by decreasing effect on the priming effect. Each functional group has a color attributed: red for A, dark blue for B, yellow for C, light blue for D, and pink for E. The above box reports the significant effects (at $p < 0.001$) of the different functional groups and their interactions. (**b**) Mean observed priming effect of soils containing a given class of family relative abundance. The linked points correspond to the different classes of increasing relative abundance (from left to right) for each family. The used symbols are the same as those used in (**a**). (**c**) Boxplots of the observed priming effect sorted by assembly motifs, i.e., combinations of functional groups. The used symbols are the same as those used in (**b**). (**d**) Modeled versus observed priming effect. Different symbols correspond to different assembly motifs, i.e., combinations of functional groups. The red line is the bisector. (**b**), (**c**) and (**d**) The dotted line is the mean observed priming effect.
